# Cardiac adverse event signals of Bruton’s tyrosine kinase inhibitors as primary suspect and in co-reported regimens: a large-scale FAERS pharmacovigilance study

**DOI:** 10.3389/fphar.2026.1923976

**Published:** 2026-10-14

**Authors:** Tingting Zhang, Ganggang Wang, Xuejing Yang, Li Li, Dong Song

**Affiliations:** 1 Department of Lymphoma, Cancer Center, Shanxi Bethune Hospital, Shanxi Academy of Medical Sciences, Tongji Shanxi Hospital, Third Hospital of Shanxi Medical University, Taiyuan, Shanxi, China; 2 Department of Thoracic Oncology, Cancer Center, Shanxi Bethune Hospital, Shanxi Academy of Medical Sciences, Tongji Shanxi Hospital, Third Hospital of Shanxi Medical University, Taiyuan, Shanxi, China

**Keywords:** bruton’s tyrosine kinase inhibitors, cardiac adverse events, cardio-oncology, combination therapy, pharmacovigilance, real-world evidence

## Abstract

**Background:**

Bruton’s tyrosine kinase inhibitors (BTKis) have improved outcomes in B-cell malignancies, but cardiac toxicity remains a clinically relevant concern, particularly with newer-generation agents and co-reported regimens. We aimed to characterize real-world cardiac adverse event signals associated with BTKis as primary suspect (PS) agents and in co-reported regimens.

**Methods:**

We conducted a pharmacovigilance study using the FDA Adverse Event Reporting System from the fourth quarter of 2013 to the first quarter of 2025. We evaluated BTKi regimens (ibrutinib, acalabrutinib, and zanubrutinib as primary suspect agents) and three specific BTKi-based co-reported combination subgroups (BTKi + anti-CD20 monoclonal antibodies, BTKi + venetoclax, and BTKi + lenalidomide). Cardiac adverse events were defined by the Medical Dictionary for Regulatory Activities “Cardiac disorders” system organ class. Disproportionality signals were assessed using four algorithms, and time-to-onset and fatal-case phenotypes were evaluated.

**Results:**

A total of 8,266 BTKi-associated cardiac adverse event reports were identified. “Cardiac disorders” showed consistent disproportionality signals across BTKis. Ibrutinib had the broadest cardiac signal spectrum, whereas acalabrutinib and zanubrutinib showed fewer but earlier-onset cardiac events. Median reported time-to-onset was 150 days for ibrutinib, 72.5 days for acalabrutinib, and 66 days for zanubrutinib. Among co-reported regimens, BTKi plus lenalidomide showed the strongest disproportional reporting signal for atrial fibrillation. Reports with fatal cardiac outcome were more frequently reported among males, patients older than 85 years, and events occurring within 30 days of treatment initiation.

**Conclusion:**

BTKis exhibit distinct real-world cardiac safety profiles. Early cardiovascular surveillance may be particularly important for newer-generation BTKis and BTKi-lenalidomide co-reported regimens, especially in patients with risk factors associated with higher reporting proportions.

## 1 Introduction

Bruton’s tyrosine kinase inhibitors (BTKis) have transformed the therapeutic landscape of B-cell malignancies by targeting B-cell receptor signaling, a central pathway in malignant B-cell survival, proliferation, and trafficking (Alu et al., 2022; Cool et al., 2024; Najmi et al., 2023). Since the introduction of the first-in-class covalent BTKi ibrutinib, subsequent next-generation agents, including acalabrutinib and zanubrutinib, have been developed with greater selectivity for BTK and reduced off-target kinase inhibition (Lewis and Cheah, 2021). These agents are now widely used across multiple B-cell malignancies, including chronic lymphocytic leukemia/small lymphocytic lymphoma, mantle cell lymphoma, marginal zone lymphoma, and Waldenström macroglobulinemia (Cool et al., 2024). However, as BTKis are often administered continuously for prolonged periods, long-term treatment-related toxicities have become increasingly relevant to clinical decision-making.

Among BTKi-associated adverse events, cardiovascular toxicities have emerged as one of the most clinically important safety concerns. Atrial fibrillation, hypertension, heart failure, ventricular arrhythmias, and sudden cardiac death have all been reported during BTKi therapy, with some events leading to treatment interruption, discontinuation, hospitalization, or death (Brown et al., 2017; Lampson et al., 2017; Salem et al., 2019; Spadafora et al., 2026). Ibrutinib-associated cardiotoxicity, including atrial fibrillation, ventricular arrhythmias, heart failure, hypertension, and sudden cardiac death, has been extensively described and is thought to be partly related to off-target inhibition of kinases involved in cardiomyocyte survival, vascular regulation, and electrophysiological stability (Salem et al., 2019). Mechanistic studies have suggested that inhibition of C-terminal Src kinase and perturbation of cardiac PI3K-Akt signaling may contribute to atrial arrhythmogenesis during BTKi exposure (McMullen et al., 2014; Xiao et al., 2020). In addition, ibrutinib also targets HER2—a critical regulator of cardiomyocyte survival, stress adaptation, and contractility, and the well-characterized cardiotoxicity target of trastuzumab (Oliva and Molica, 2026). Therefore, cardiac toxicity is no longer considered a rare or incidental complication of BTKi therapy, but rather a central challenge in contemporary cardio-oncology practice, underscoring the need for comprehensive cardiovascular risk assessment and surveillance in patients receiving these agents.

Next-generation BTKis were designed to improve target selectivity and reduce off-target toxicities, raising the expectation that they may offer improved cardiovascular safety compared with ibrutinib. This has been partly supported by two pivotal head-to-head randomized clinical trials. In ELEVATE-RR, acalabrutinib demonstrated noninferior efficacy and a lower incidence of atrial fibrillation compared with ibrutinib in patients with previously treated chronic lymphocytic leukemia (Byrd et al., 2021). Similarly, in ALPINE, zanubrutinib showed superior efficacy and a more favorable cardiac safety profile than ibrutinib, including a lower rate of atrial fibrillation or flutter (Brown et al., 2023). Nevertheless, cardiovascular adverse events were not eliminated with either acalabrutinib or zanubrutinib. Moreover, randomized trials are limited by selected eligibility criteria, protocol-driven monitoring, and relatively constrained sample sizes for uncommon but severe cardiovascular outcomes. Thus, important questions remain regarding the real-world spectrum, timing, and clinical severity of cardiac adverse events associated with different BTKi generations.

In parallel, the treatment paradigm for B-cell malignancies is rapidly evolving from single-agent BTKi therapy toward combination regimens designed to achieve deeper and more durable remissions. BTKis are increasingly combined with anti-CD20 monoclonal antibodies, BCL-2 inhibitors such as venetoclax, or immunomodulatory agents such as lenalidomide (Shanafelt et al., 2019; Tam et al., 2022; Zhu et al., 2022). Although these combinations have demonstrated promising antitumor activity, their additive or synergistic cardiac toxicity profiles remain incompletely characterized. This gap is particularly important because lenalidomide has been associated with an increased risk of thrombotic events, which may interact with BTKi-related cardiovascular risk (Palumbo et al., 2008). The combination of vascular stress, prothrombotic tendency, and BTKi-related atrial vulnerability may therefore create a clinically relevant substrate for amplified cardiac risk. However, large-scale real-world evidence evaluating cardiac safety signals across BTKi-based combinations remains limited.

Current cardio-oncology guidelines emphasize baseline cardiovascular risk assessment, surveillance during cancer therapy, and individualized management of treatment-related cardiac toxicity (Lyon et al., 2022). However, specific monitoring strategies for different BTKi agents and BTKi-based combination regimens are still not well defined, especially regarding the optimal timing of electrocardiographic surveillance and identification of patients at highest risk for fatal cardiac events. Specifically: (i) although cardiac events with next-generation BTKis appear to cluster earlier in treatment, guidelines have not yet incorporated this temporal pattern into differentiated early monitoring recommendations; (ii) whether BTKi-based co-reported regimens confer an enhanced arrhythmic risk remains uncharacterized by real-world safety data; and (iii) which patient characteristics (age, sex, body weight, timing of onset) are associated with cardiac adverse events is largely unknown. Real-world pharmacovigilance data can complement clinical trial evidence by capturing broader patient populations, rare adverse events, early post-marketing signals, and severe outcomes that may be underrepresented in randomized trials.

To address these clinically important gaps, we conducted a large-scale pharmacovigilance study using the FDA Adverse Event Reporting System database to systematically characterize disproportional cardiac adverse event reporting signals associated with BTKi therapy. We evaluated ibrutinib, acalabrutinib, and zanubrutinib as the primary suspect (PS) agents, as well as contemporary BTKi-based co-reported regimens. By integrating disproportionality analysis, time-to-onset assessment, and fatal-case phenotyping, this study aimed to characterize agent-specific and regimen-specific cardiac safety signal profiles and to provide clinically relevant insights for individualized cardio-oncology surveillance.

## 2 Materials and methods

### 2.1 Data source and case identification

This retrospective pharmacovigilance study utilized data extracted from the FAERS database. To ensure robust signal detection, we aggregated quarterly data files from the fourth quarter of 2013 to the first quarter of 2025. According to the guidelines of the FAERS database, duplicate adverse event reports were removed: (1) if CASEID matched, the latest FDA_DT was selected; (2) if CASEID and FDA_DT were the same, the higher PRIMARYID was chosen, ensuring that only the most recent version of each unique case was retained for analysis. To differentiate between PS-based BTKi reports and co-reported concomitant subgroups, we further examined all concomitant drugs reported within the same PRIMARYID. Specifically, for each BTKi-associated report (with BTKi as PS), the presence of other drugs—including rituximab, obinutuzumab, venetoclax, or lenalidomide—was identified from the concomitant (C), interacting (I), or secondary suspect (SS) drug roles. A report was classified as a co-reported concomitant regimen if at least one of these agents was co-reported with the BTKi. The specific co-reported subgroups were defined as BTKi + anti-CD20 monoclonal antibodies (rituximab/obinutuzumab), BTKi + venetoclax, and BTKi + lenalidomide. To ensure comprehensive capture of all relevant reports, we created drug name dictionaries for each BTKi and each co-reported agent, encompassing brand names, generic names, research codes, and common variations (Supplementary Table S1). All drug names were standardized using the RxNorm, and adverse event reports enrolled in this study were standardized and categorized at the system organ class (SOC) and preferred term (PT) levels utilizing the MedDRA (version 27.1).

### 2.2 Signal detection and disproportionality analysis

To mitigate reporting biases inherent in spontaneous reporting systems, we employed four validated disproportionality algorithms: the Reporting Odds Ratio (ROR), Proportional Reporting Ratio (PRR), Bayesian Confidence Propagation Neural Network (BCPNN), and Multi-item Gamma Poisson Shrinker (MGPS). Associations between drug exposure and adverse events were analyzed through these algorithms based on a four-grid table (Supplementary Table S2), and the specific formula and threshold for each algorithm were displayed in Supplementary Table S3 (Liu et al., 2024). A positive cardiac safety signal was defined only when all four algorithms simultaneously met their respective conservative significance thresholds. This stringent approach ensures that the identified signals represent meaningful clinical associations rather than random statistical noise (Caster et al., 2020; Zhou et al., 2023).

### 2.3 Time-to-onset and survival analysis

The time to adverse event occurrence refers to the time interval between the start of medication (START_DT) and the time when the adverse event occurs (EVENT_DT). After removing reports with inaccurate date, missing data, or EVENT_DT earlier than START_DT, TTO distribution was described by median, interquartile range, extreme value (minima and maxima), and Weibull distribution modeling. To compare TTO distributions across different drug groups, we constructed Kaplan-Meier curves and performed log-rank tests, with *P* < 0.05 considered statistically significant. In Weibull distribution modeling, a β < 1 indicates an early failure type (risk decreases over time), a β > 1 suggests cumulative toxicity, and β ≈ 1 demonstrated a constant risk (random failure pattern).

### 2.4 Patient phenotyping and multivariable analysis

To identify report-level factors associated with cardiac adverse event reporting, multivariable logistic regression models were constructed. The outcome was defined as the reporting of at least one PT within the “Cardiac disorders” SOC. Covariates included age (<18, 18–65, 65–85, and ≥85), sex (female and male), body weight (<80, 80–100, and ≥100), and drugs (zanubrutinib, acalabrutinib, and ibrutinib). A complete case analysis was applied, excluding reports with missing data for any of the covariates. Collinearity was assessed using variance inflation factors, with values <5 indicating no significant multicollinearity. Results were expressed as adjusted odds ratios (aORs) with 95% confidence intervals (CIs). All odds ratios represent associations at the level of FAERS reporting and should not be interpreted as patient-level clinical risk factors. Statistical analyses were performed using R software (version 4.2.1), with *P*-values <0.05 considered statistically significant.

## 3 Results

### 3.1 Descriptive characteristics of the study cohort

A large-scale cohort of BTKi-associated adverse event reports was systematically identified from the FAERS database. The detailed flow of adverse event report selection and data deduplication is illustrated in Supplementary Figure S1. FDA-approved BTK inhibitors included in the final analysis were ibrutinib, acalabrutinib, and zanubrutinib, whereas newly approved agents with limited exposure were excluded (Supplementary Table S4).

For BTKi PS-based cohorts (ibrutinib, acalabrutinib, and zanubrutinib), the demographic baseline prominently featured older adults and a male predominance, broadly consistent with the epidemiological landscape of B-cell malignancies (Figure 1; Supplementary Table S5). Detailed distribution patterns regarding age, sex, weight, outcomes, and reporter occupations across the three PS-based groups are presented in Supplementary Figure S2. Similarly, distinct baseline characteristics and temporal reporting trends were established for co-reported combination regimens, including BTKi plus venetoclax, obinutuzumab/rituximab, and lenalidomide (Supplementary Table S6; Supplementary Figure S3).

**FIGURE 1:**
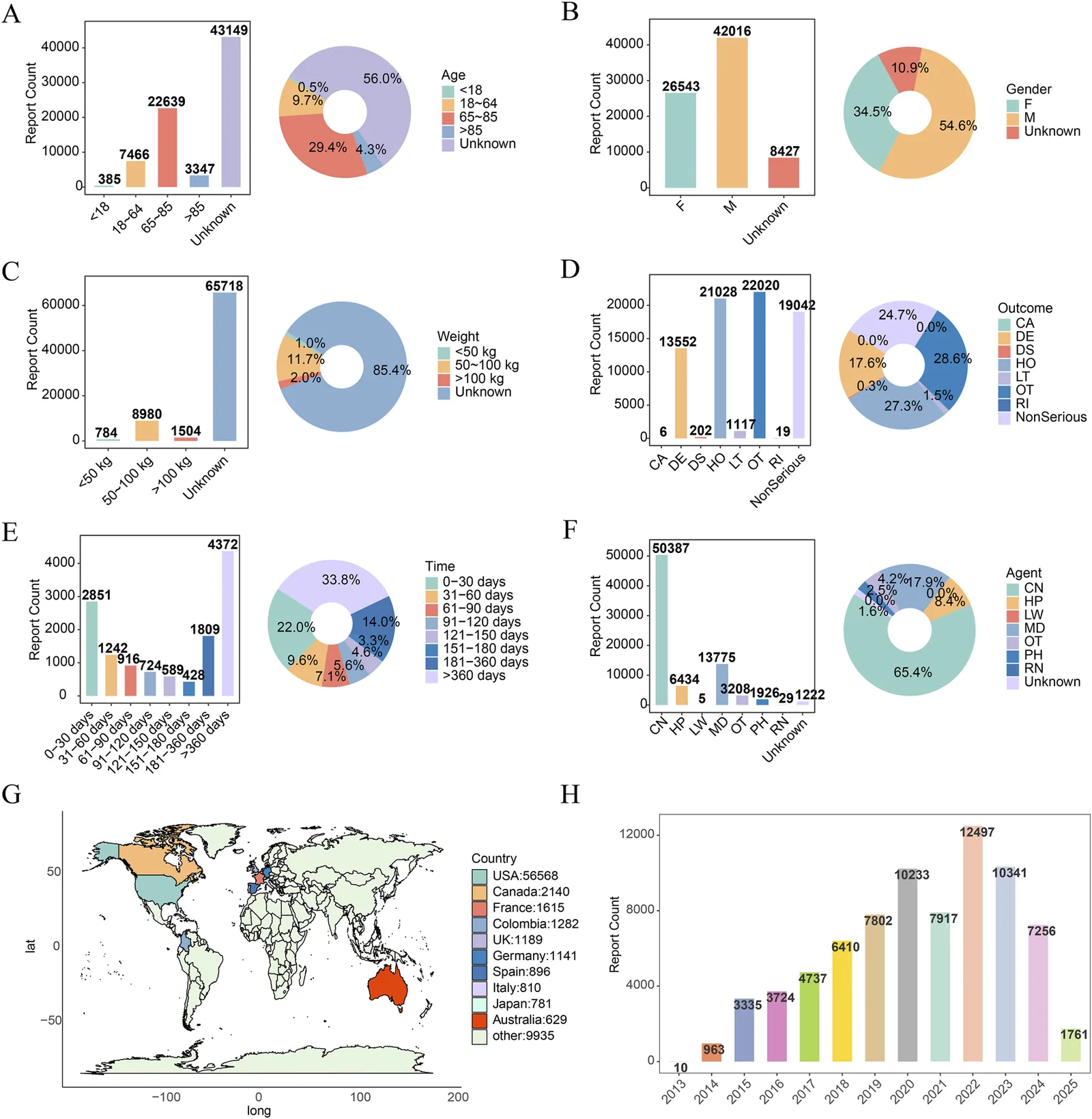
Baseline characteristics of adverse event (AE) reports associated with Bruton’s tyrosine kinase inhibitor (BTKi) therapy **(A)** Age **(B)** Sex **(C)**. Body weight. **(D)** Reported outcomes: CA, congenital anomaly; DE, death; DS, disability; HO, hospitalization; LT, life-threatening; OT, other serious outcomes; RI, required intervention. **(E)** Time to AE occurrence. **(F)** Reporter occupation: CN, consumer; HP, health professional; LW, lawyer; MD, physician; OT, other health professional; PH, pharmacist; RN, registered nurse. **(G)** Reporting country. **(H)** Annual distribution of reports.

### 3.2 Overall pharmacovigilance signal landscape

Disproportionality analysis was anchored by a robust two-by-two contingency framework utilizing four complementary signal detection algorithms (ROR, PRR, BCPNN, and MGPS). At the PT level, the analysis encompassed 5,958 unique PTs spanning 27 SOCs (Figure 2A). Using the ROR algorithm, positive disproportionality signals were initially detected for 9 SOCs, including “Cardiac disorders,” “Blood and lymphatic system disorders,” and “Infections and infestations” (Figure 2B). To ensure robustness, three additional algorithms (PRR, BCPNN, and MGPS) were applied. Notably, three SOCs—“Various surgical and medical procedures,” “Cardiac disorders,” and “Blood and lymphatic system disorders”—consistently generated positive signals across all four algorithms, demonstrating consistent disproportionality in association with BTKi therapy (Figure 2C). Given its clinical relevance and the consistency of the signal, the SOC “Cardiac disorders” was selected for further investigation.

**FIGURE 2:**
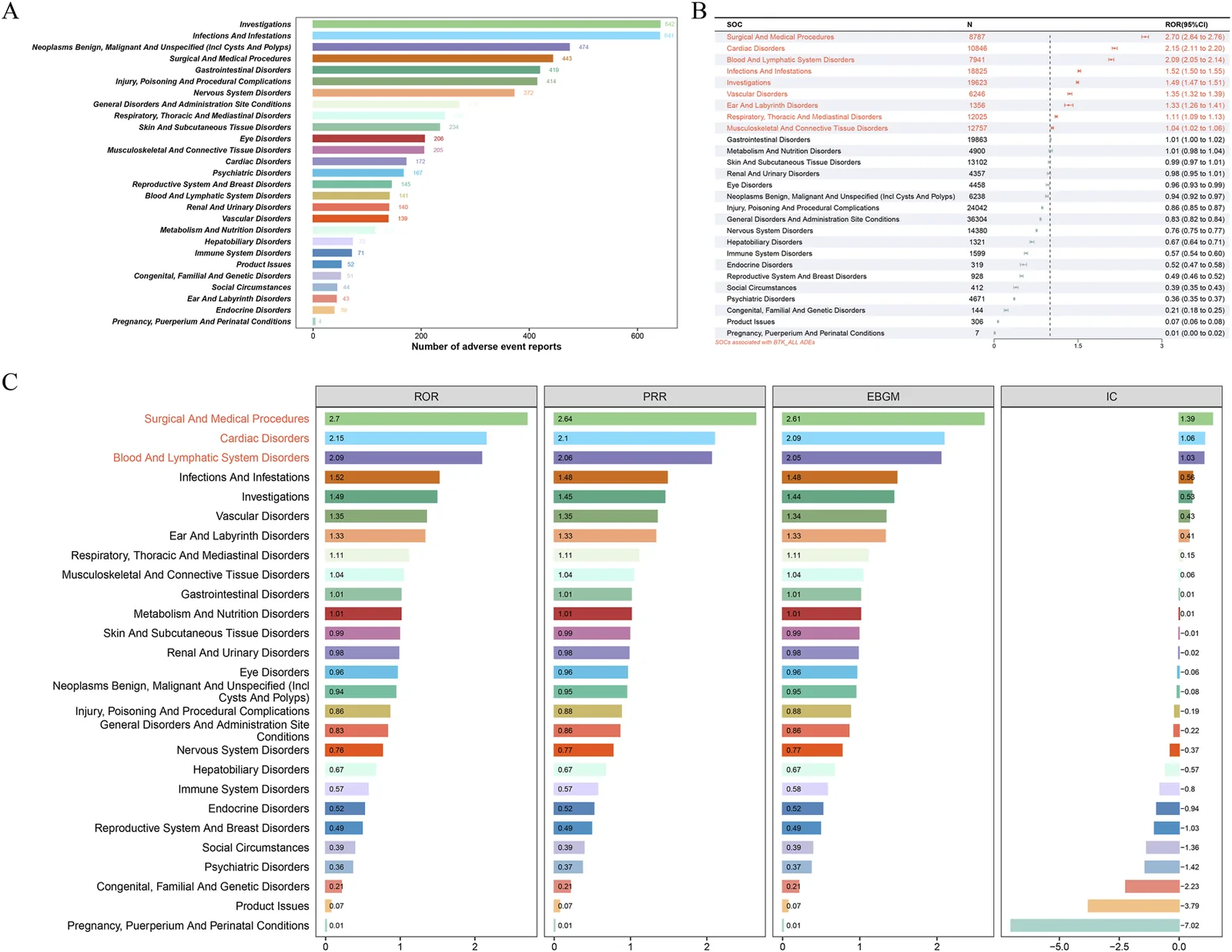
Distribution of adverse event reports associated with BTKi therapy at the System Organ Class (SOC) level. **(A)** Number of AE reports in each SOC. **(B)** Forest plot of reporting odds ratio (ROR) values for each SOC; red indicates SOCs meeting the ROR signal threshold. **(C)** Pharmacovigilance signal strength of SOCs evaluated using ROR, proportional reporting ratio (PRR), information component (IC), and empirical Bayesian geometric mean (EBGM); red indicates SOCs meeting the signal thresholds of all four algorithms.

### 3.3 Analysis of cardiac disorder cases

Building on the signal detection results that consistently identified “Cardiac disorders” as a significant SOC, we next characterized these specific adverse events in greater detail. Among the overall BTKi-exposed population, 8,266 reports involved cardiac adverse events, with most reports in the ibrutinib PS-based group (*n* = 7,404), followed by acalabrutinib (*n* = 718) and zanubrutinib (*n* = 144) (Figure 3A). Among the co-reported combination subgroups, BTKi plus obinutuzumab/rituximab had the highest number of cardiac adverse event reports (*n* = 423), followed by BTKi plus venetoclax (*n* = 249) and BTKi plus lenalidomide (*n* = 52) (Figure 3A). The baseline characteristics of these reports are summarized in Table 1.

**FIGURE 3:**
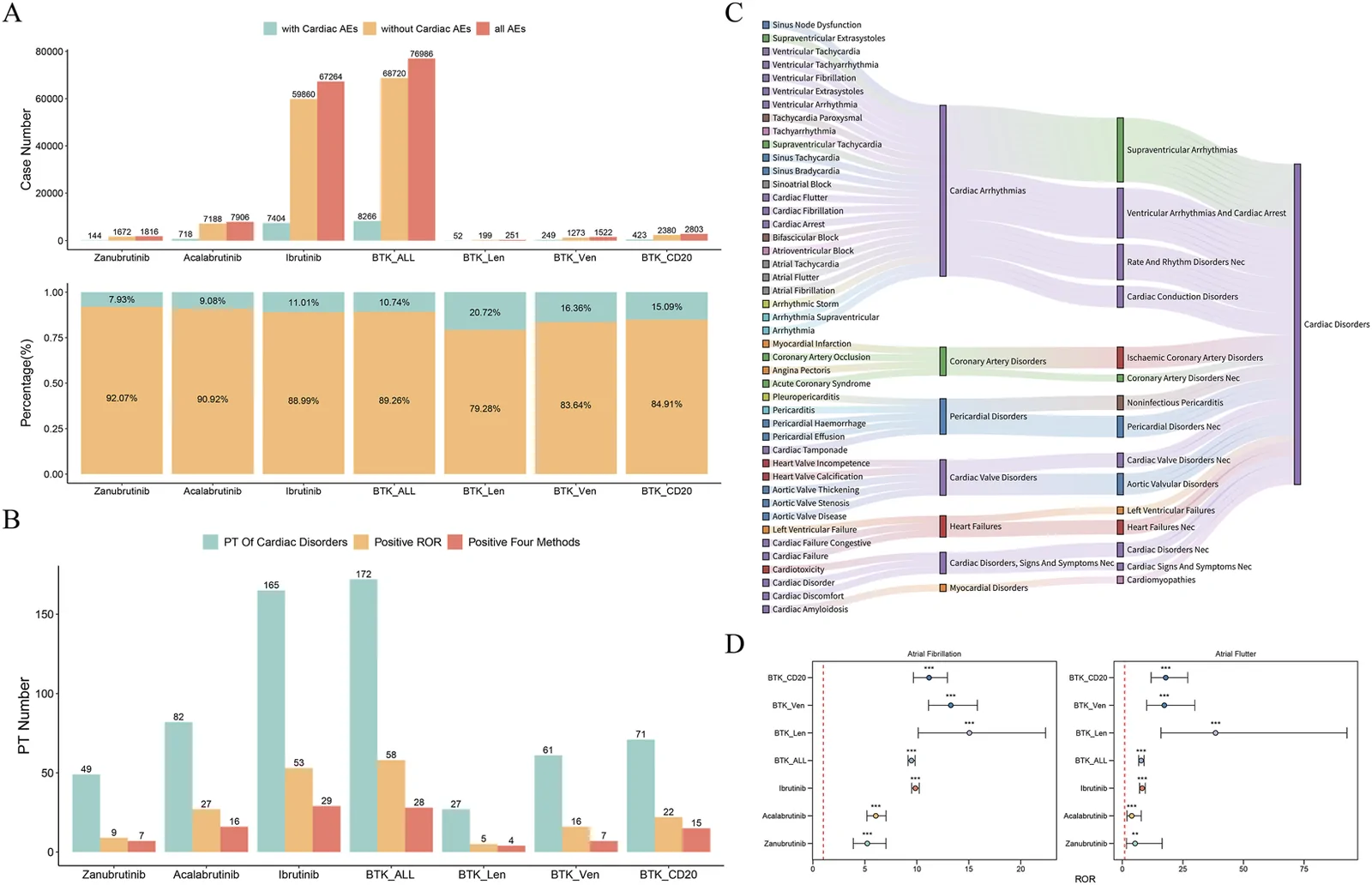
Cardiac adverse event reporting profiles across different BTKi reporting groups. **(A)** Proportion of AE reports involving cardiac adverse events in each reporting group. **(B)** Number of positive cardiac AE signals at the Preferred Term (PT) level in each reporting group. **(C)** Classification of cardiac AEs across all reporting groups after deduplication. **(D)** ROR values for common cardiac AEs across reporting groups. **P < 0.01, ***P < 0.001.

**TABLE 1:** Baseline characteristics of cardiac adverse event reports associated with Bruton’s tyrosine kinase inhibitor (BTKi) therapy.

	BTKi				BTKi combination regimens		
Characteristics	All	Acalabrutinib	Ibrutinib	Zanubrutinib	BTKi + venetoclax	BTKi + obinutuzumab/rituximab	BTKi + lenalidomide
​	(*N* = 8,266)	(*N* = 718)	(*N* = 7,404)	(*N* = 144)	(*N* = 249)	(*N* = 423)	(*N* = 52)
Sex							
Female	2,380 (28.8%)	174 (24.2%)	2,205 (29.8%)	1 (0.7%)	44 (17.7%)	101 (23.9%)	13 (25.0%)
Male	4,669 (56.5%)	319 (44.4%)	4,346 (58.7%)	4 (2.8%)	149 (59.8%)	244 (57.7%)	25 (48.1%)
Unknown	1,217 (14.7%)	225 (31.3%)	853 (11.5%)	139 (96.5%)	56 (22.5%)	78 (18.4%)	14 (26.9%)
Body weight							
<50 kg	78 (0.9%)	5 (0.7%)	72 (1.0%)	1 (0.7%)	3 (1.2%)	6 (1.4%)	2 (3.8%)
>100 kg	276 (3.3%)	32 (4.5%)	244 (3.3%)	—	12 (4.8%)	34 (8.0%)	3 (5.8%)
50–100 kg	1,276 (15.4%)	128 (17.8%)	1,142 (15.4%)	6 (4.2%)	46 (18.5%)	125 (29.6%)	12 (23.1%)
Unknown	6,636 (80.3%)	553 (77.0%)	5,946 (80.3%)	137 (95.1%)	188 (75.5%)	258 (61.0%)	35 (67.3%)
Age							
<18	42 (0.5%)	—	42 (0.6%)	​	2 (0.8%)	1 (0.2%)	​
>85	412 (5.0%)	27 (3.8%)	384 (5.2%)	1 (0.7%)	3 (1.2%)	13 (3.1%)	3 (5.8%)
18–64	831 (10.1%)	47 (6.5%)	782 (10.6%)	2 (1.4%)	46 (18.5%)	91 (21.5%)	8 (15.4%)
65–85	3,165 (38.3%)	173 (24.1%)	2,981 (40.3%)	11 (7.6%)	97 (39.0%)	207 (48.9%)	25 (48.1%)
Unknown	3,816 (46.2%)	471 (65.6%)	3,215 (43.4%)	130 (90.3%)	101 (40.6%)	111 (26.2%)	16 (30.8%)
Reporter occupation							
Consumer	4,297 (52.0%)	329 (45.8%)	3,928 (53.1%)	40 (27.8%)	61 (24.5%)	78 (18.4%)	8 (15.4%)
Health-professional	728 (8.8%)	39 (5.4%)	673 (9.1%)	16 (11.1%)	32 (12.9%)	58 (13.7%)	5 (9.6%)
Physician	2,321 (28.1%)	231 (32.2%)	2024 (27.3%)	66 (45.8%)	140 (56.2%)	254 (60.0%)	34 (65.4%)
Other health-professional	522 (6.3%)	1 (0.1%)	521 (7.0%)	—	13 (5.2%)	18 (4.3%)	2 (3.8%)
Pharmacist	275 (3.3%)	17 (2.4%)	243 (3.3%)	15 (10.4%)	3 (1.2%)	8 (1.9%)	2 (3.8%)
Registered nurse	2 (0.0%)	—	2 (0.0%)	—	—	—	​
Unknown	121 (1.5%)	101 (14.1%)	13 (0.2%)	7 (4.9%)	—	7 (1.7%)	1 (1.9%)
Serious outcomes							
Congenital anomaly	1 (0.0%)	1 (0.1%)	—	—	—	—	—
Death	1,125 (13.6%)	53 (7.4%)	1,053 (14.2%)	19 (13.2%)	36 (14.5%)	76 (18.0%)	14 (26.9%)
Disability	9 (0.1%)	1 (0.1%)	8 (0.1%)	—	—	—	—
Hospitalization	3,468 (42.0%)	260 (36.2%)	3,162 (42.7%)	46 (31.9%)	89 (35.7%)	202 (47.8%)	23 (44.2%)
Life-threatening	311 (3.8%)	41 (5.7%)	262 (3.5%)	8 (5.6%)	16 (6.4%)	36 (8.5%)	3 (5.8%)
Other serious outcomes	2,558 (30.9%)	306 (42.6%)	2,189 (29.6%)	63 (43.8%)	93 (37.3%)	96 (22.7%)	11 (21.2%)
Required intervention	3 (0.0%)	—	3 (0.0%)	—	—	—	—
Missing	791 (9.6%)	56 (7.8%)	727 (9.8%)	8 (5.6%)	15 (6.0%)	13 (3.1%)	1 (1.9%)
Top 5 reporting countries							
​	United state (5,364, 64.9%)	United state (500, 69.6%)	United state (4,794, 64.7%)	United state (70, 48.6%)	United state (111, 44.6%)	United state (203, 48.0%)	United state (35, 67.3%)
​	France (304, 3.7%)	United kindom (30, 4.2%)	France (269, 3.6%)	China (11, 7.6%)	Germany (20, 8.0%)	United kindom (22, 5.2%)	China (4, 7.7%)
​	Canada (241, 2.9%)	Germany (29, 4.0%)	Canada (209, 2.8%)	Canada (10, 6.9%)	Italy (13, 5.2%)	Germany (20, 4.7%)	France (4, 7.7%)
​	Germany (209, 2.5%)	France (29, 4.0%)	Spain (187, 2.5%)	Australia (9, 6.3%)	United kindom (12, 4.8%)	France (17, 4.0%)	United kindom (3, 5.8%)
​	Spain (194, 2.3%)	Canada (22, 3.1%)	United kindom (149, 2.0%)	Germany (6, 4.2%)	Australia (10, 4.0%)	Australia (16, 3.8%)	Greece (2, 3.8%)
​	​	​	​	France (6, 4.2%)	​	​	Sweden (2, 3.8%)
Reporting year							
2014	139 (1.7%)	—	139 (1.9%)	—	—	2 (0.5%)	​
2015	402 (4.9%)	—	402 (5.4%)	—	—	16 (3.8%)	​
2016	507 (6.1%)	—	507 (6.8%)	—	—	18 (4.3%)	2 (3.8%)
2017	653 (7.9%)	7 (1.0%)	646 (8.7%)	—	3 (1.2%)	42 (9.9%)	7 (13.5%)
2018	796 (9.6%)	12 (1.7%)	784 (10.6%)	—	8 (3.2%)	44 (10.4%)	10 (19.2%)
2019	912 (11.0%)	5 (0.7%)	904 (12.2%)	3 (2.1%)	37 (14.9%)	34 (8.0%)	7 (13.5%)
2020	1,126 (13.6%)	26 (3.6%)	1,094 (14.8%)	6 (4.2%)	52 (20.9%)	90 (21.3%)	9 (17.3%)
2021	819 (9.9%)	59 (8.2%)	751 (10.1%)	9 (6.3%)	39 (15.7%)	50 (11.8%)	2 (3.8%)
2022	950 (11.5%)	129 (18.0%)	803 (10.8%)	18 (12.5%)	22 (8.8%)	37 (8.7%)	2 (3.8%)
2023	938 (11.3%)	146 (20.3%)	756 (10.2%)	36 (25.0%)	30 (12.0%)	42 (9.9%)	6 (11.5%)
2024	836 (10.1%)	281 (39.1%)	494 (6.7%)	61 (42.4%)	33 (13.3%)	36 (8.5%)	3 (5.8%)
2025	188 (2.3%)	53 (7.4%)	124 (1.7%)	11 (7.6%)	25 (10.0%)	12 (2.8%)	4 (7.7%)

At the PT level, using the stringent criterion of simultaneous positivity across all four algorithms, we identified 29, 16, and seven positive cardiac signals for ibrutinib, acalabrutinib, and zanubrutinib, respectively (Figure 3B; Supplementary Figure S4; Supplementary Table S7). In the co-reported combination subgroups, 15, 7, and four positive signals were detected for BTKi plus obinutuzumab/rituximab, BTKi plus venetoclax, and BTKi plus lenalidomide, respectively (Figure 3B; Supplementary Figure S5; Supplementary Table S8). After deduplication, 45 unique cardiac PTs were identified (Figure 3C; Supplementary Table S9). Atrial fibrillation and atrial flutter emerged as commonly reported significant adverse events across all treatment groups. The ROR values for these events varied substantially, with the BTKi plus lenalidomide subgroup showing the strongest disproportionality signal (atrial fibrillation: ROR = 15.06, 95% CI = 10.13–22.39; atrial flutter: ROR = 38.49, 95% CI = 15.98–92.69) (Figure 3D). Notably, because BTKIs were pooled as a class in this combination analysis and the lenalidomide subgroup included a mixture of various BTKIs, this signal should be interpreted as preliminary and hypothesis-generating, rather than evidence of true synergy.

### 3.4 TTO analysis of cardiac adverse events

The reported TTO of cardiac adverse events appeared to differ among BTK inhibitors. Among reports with evaluable dates, the median TTO was observed to be shorter for the second-generation inhibitors acalabrutinib (median, 72.5 days; IQR, 15–182.75) and zanubrutinib (median, 66 days; IQR, 16.5–222.5) than for the first-generation agent ibrutinib (median, 150 days; IQR, 42–479) (Supplementary Table S10; Figures 4A–C). This pattern was also reflected in the cumulative distribution curves, which showed a significant difference across the three PS-based groups (*P* < 0.001; Figure 4D).

**FIGURE 4:**
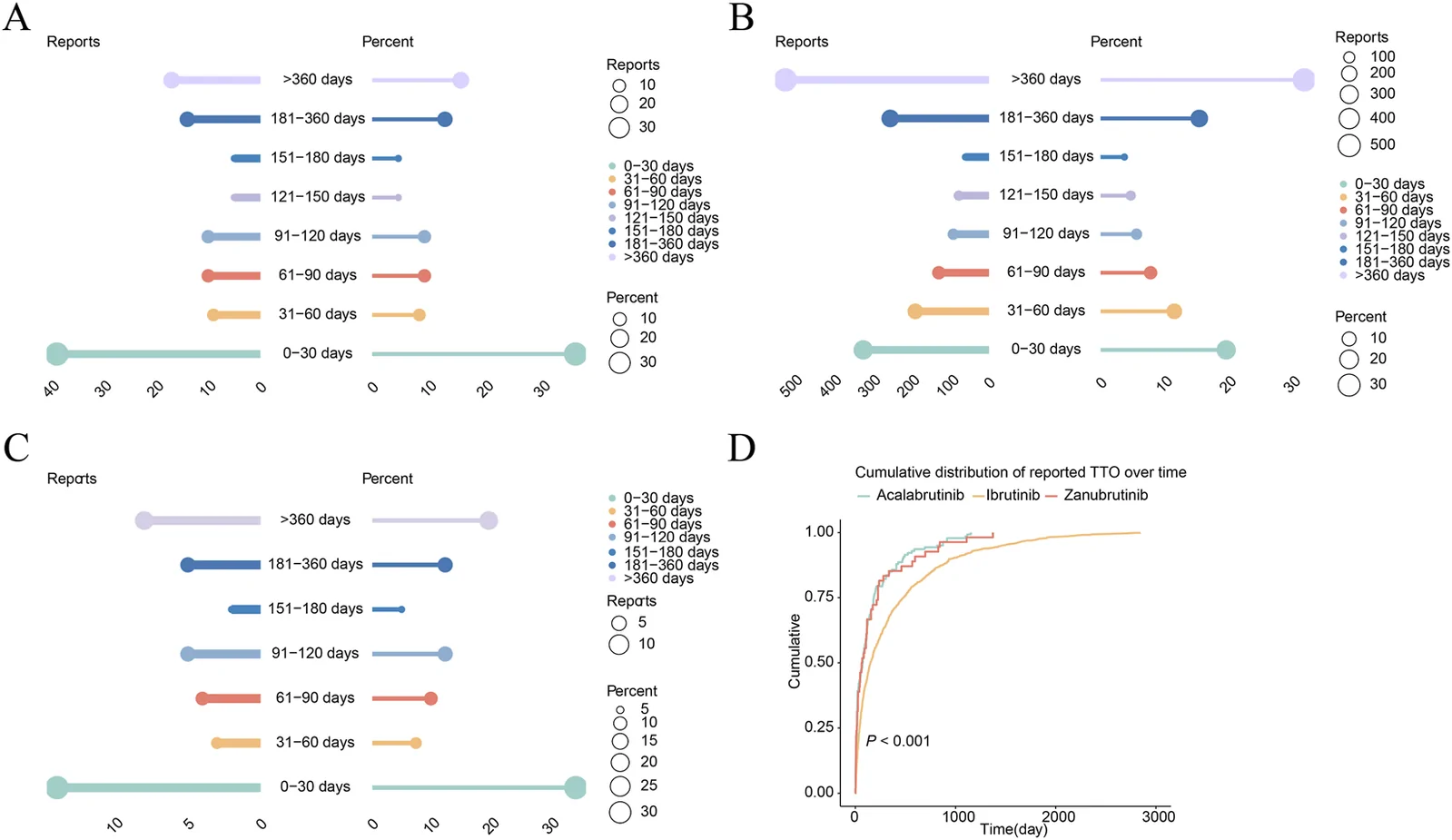
Time-to-onset characteristics of reported cardiac adverse events across different PS-based reporting groups. **(A)** Acalabrutinib PS-based group. **(B)** Ibrutinib PS-based group. **(C)** Zanubrutinib PS-based group. **(D)** Comparison of cumulative distribution of reported time-to-onset over time among the three BTKi PS-based cohorts.

In the co-reported combination subgroups, the median TTO also varied, being 180.5 days for BTKi plus venetoclax, 81 days for BTKi plus obinutuzumab/rituximab, and 34 days for BTKi plus lenalidomide (Supplementary Table S11; Figures 5A–C). However, the cumulative distribution curves did not differ significantly among the co-reported subgroups (*P* = 0.2; Figure 5D). Weibull distribution modeling indicated that most groups exhibited an “early failure” pattern (β < 1), suggesting that reported events were more likely to be reported earlier in treatment. An exception was the BTKi plus venetoclax subgroup, in which the reporting pattern appeared more constant over time (β ≈ 1) (Supplementary Table S11). It should be noted, however, that these TTO findings are exploratory, as FAERS TTO distributions may be influenced by stimulated reporting, the Weber effect, and differential exposure durations across agents.

**FIGURE 5:**
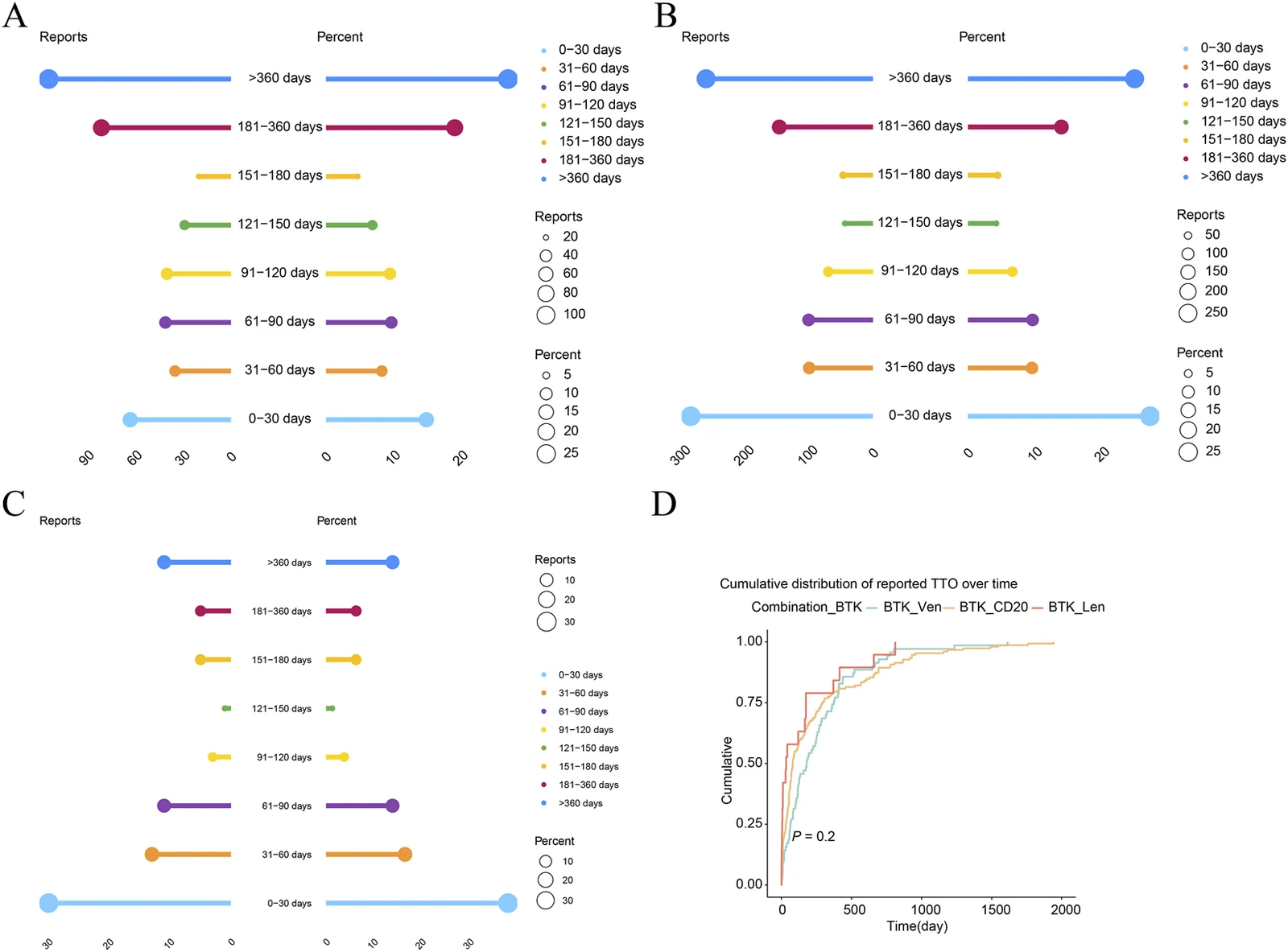
Time-to-onset characteristics of reported cardiac adverse events across different co-reported groups. **(A)** BTKi plus venetoclax subgroup. **(B)** BTKi plus obinutuzumab/rituximab subgroup. **(C)** BTKi plus lenalidomide subgroup. **(D)** Comparison of cumulative distribution of reported time-to-onset over time among the BTKi-based co-reported subgroups.

### 3.5 Comparison between cardiac adverse events with stratified by fatal outcome status

A death outcome was the most severe outcome among reports involving cardiac adverse events during BTKi therapy. The baseline characteristics of reports with fatal and non-fatal cases are summarized in Supplementary Table S12. To ensure statistically robust subgroup comparisons, cases with “Unknown” values were excluded from subsequent analyses. The group of reports with fatal outcome exhibited a distinct reporting profile, including a higher proportion of males (75.8% vs. 64.8%; Figure 6A) and a greater percentage of patients weighing <50 kg (Figure 6B). A marked age disparity was also observed, with nearly twice as many individuals aged >85 years in the reports with fatal outcome group (15.0% vs. 8.2%; Figure 6C). Reporting patterns also differed substantially: fatal outcomes were more often reported by physicians (39.0% vs. 26.8%), whereas non-fatal cases were more commonly reported by consumers (56.3% vs. 30.6%; Figure 6D). Ibrutinib accounted for most fatal reports (93.6% vs. 88.9%), whereas zanubrutinib was associated with fewer fatal reports (1.8% vs. 1.7%; Figure 6E). Temporally, reports with fatal outcome tended to be reported earlier, with 25.8% reported within 30 days of treatment initiation compared with 19.9% in non-fatal cases (Figure 6F). All comparisons in this section are descriptive and have not been adjusted for potential confounders; therefore, they should not be interpreted as establishing risk factors for fatal outcomes.

**FIGURE 6:**
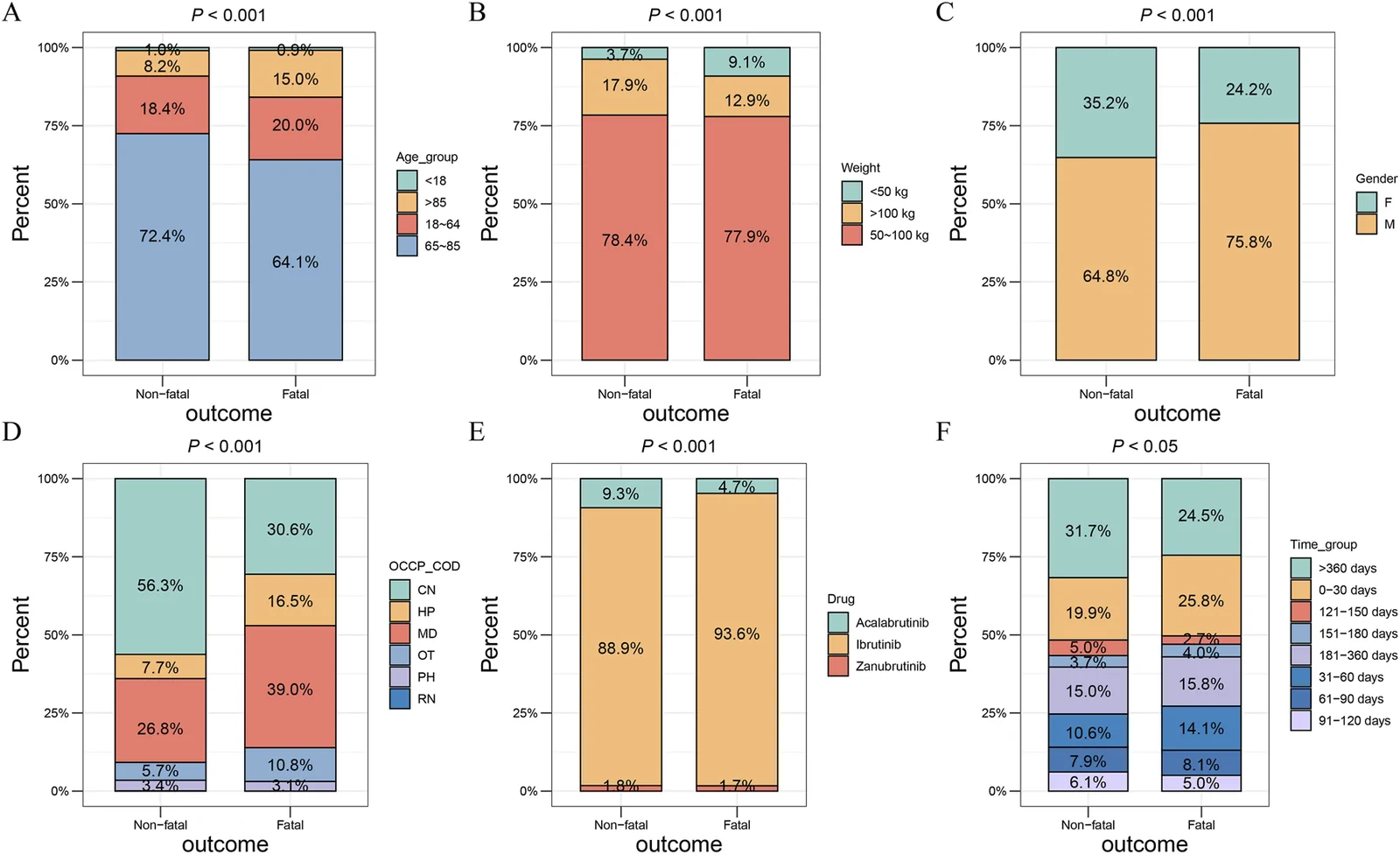
Differences in baseline report characteristics of cardiac adverse event reports stratified by fatal outcome status. **(A)** Age. **(B)** Body weight. **(C)** Sex. **(D)** Reporter occupation. **(E)** Primary suspect BTKi regimen. **(F)** Time to onset of cardiac adverse events.

### 3.6 Factors associated with reporting BTKi-related cardiac adverse events

Univariate and multivariable logistic regression analyses were performed to explore report-level factors associated with BTKi-related cardiac adverse events. Collinearity diagnostics indicated no significant multicollinearity among the included covariates, with all variance inflation factor values below 5 (Supplementary Table S13). In univariate analysis (Figure 7A), body weight (80–100 and ≥100), male sex, and BTKi agent (ibrutinib) were significantly associated with the reporting odds of cardiac adverse events (*P* < 0.05). Multivariable logistic regression was performed on a complete-case sample of 8,623 reports (with non-missing data for all covariates included in the model). Results showed that higher body weight (80–100 and ≥100) remained significantly associated with increased reporting odds of cardiac adverse events (Figure 7B). In contrast, the associations observed for male sex and ibrutinib use in univariate analyses were attenuated after adjustment. All odds ratios represent associations at the level of FAERS reporting and should not be interpreted as patient-level clinical risk factors.

**FIGURE 7:**
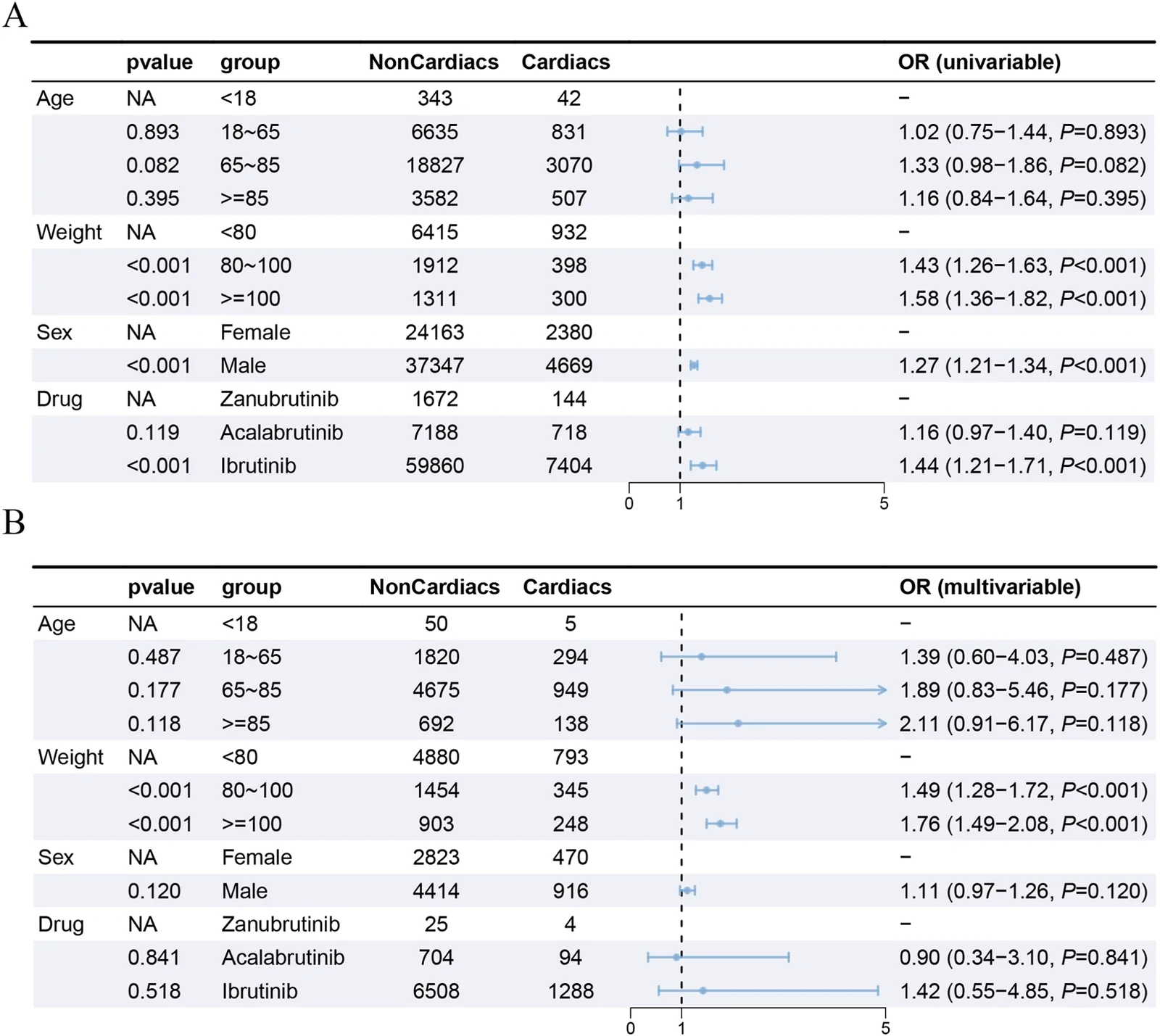
Report-level factors associated with cardiac adverse event reporting in BTKi-related reports. **(A)** Univariate logistic regression analysis. **(B)** Multivariable logistic regression analysis.

## 4 Discussion

As the therapeutic application of BTKis expands in hematologic oncology, managing their cardiovascular toxicities has become an increasingly important clinical challenge. To our knowledge, this is one of the largest real-world pharmacovigilance studies systematically delineating cardiac adverse event reporting signals across BTKi PS-based cohorts and contemporary co-reported combination subgroups. By integrating disproportionality analysis, time-to-onset characterization, and fatal-case phenotyping, our study provides clinically meaningful insights for signal-based stratification and surveillance.

Consistent with previous clinical and pharmacovigilance evidence, our analysis identified that cardiotoxicity represents a class-related safety concern of BTK inhibition, although the reporting profiles differed across individual agents (Zheng et al., 2023). Ibrutinib exhibited the broadest spectrum of cardiac adverse event signals, which is biologically plausible given its well-documented off-target inhibition of kinases involved in myocardial homeostasis and electrophysiological regulation. Mechanistic studies have implicated disruption of PI3K-Akt signaling and inhibition of C-terminal Src kinase as potential contributors to ibrutinib-associated atrial fibrillation (McMullen et al., 2014; Xiao et al., 2020). In contrast, acalabrutinib and zanubrutinib were developed to improve BTK selectivity and reduce off-target toxicity (Brown et al., 2023; Byrd et al., 2021).

Importantly, our real-world findings are broadly concordant with major head-to-head randomized trials, and also add a complementary perspective to their clinical interpretation. In ELEVATE-RR, acalabrutinib demonstrated a lower incidence of atrial fibrillation than ibrutinib in previously treated chronic lymphocytic leukemia, although cardiovascular events were not completely eliminated (Byrd et al., 2021). Similarly, in ALPINE, zanubrutinib showed superior efficacy and a more favorable cardiac safety profile compared with ibrutinib, including a lower rate of atrial fibrillation or flutter (Brown et al., 2023). Our FAERS analysis aligns with these randomized data, showing that while ibrutinib was associated with the largest number of positive cardiac PT signals, acalabrutinib and zanubrutinib also exhibited distinct cardiac reporting profiles. Nevertheless, comparisons of signal counts across agents should be interpreted with caution, as they are influenced by differential drug exposure durations and reporting volumes in FAERS. However, our study adds a clinically important observation not readily captured by conventional trial safety summaries: cardiac adverse events reported with acalabrutinib and zanubrutinib appeared to have a shorter TTO than those with ibrutinib.

The TTO findings may have implications for clinical surveillance, although they should be interpreted with caution. We observed that the median reported onset of cardiac adverse events was approximately 150 days for ibrutinib, compared with approximately 66–72.5 days for zanubrutinib and acalabrutinib. Weibull modeling further suggested an early-failure pattern for most BTKi groups, indicating that reported cardiac events were more likely to cluster during the early treatment period. This early reporting pattern could hypothetically reflect acute electrophysiological susceptibility, unmasking of subclinical cardiovascular disease, early blood pressure destabilization, or interaction with pre-existing arrhythmogenic substrates, although these possibilities remain speculative and cannot be confirmed by FAERS data. Nevertheless, it is important to acknowledge that TTO distributions in FAERS may be influenced by stimulated reporting, the Weber effect, and differential drug exposure durations across agents. Therefore, these findings should be considered exploratory and hypothesis-generating rather than definitive evidence of earlier cardiotoxicity. Nonetheless, they underscore the importance of early cardiovascular monitoring even with next-generation BTKis.

A particularly novel finding of our study concerns BTKi-based co-reported regimens. Although combinations such as BTKi plus venetoclax have demonstrated manageable safety profiles in clinical trials and selected clinical settings (Castillo et al., 2026; Tam et al., 2022), our analysis identified a notably strongly disproportionality signal for atrial fibrillation and atrial flutter when BTKis were co-reported with lenalidomide (atrial fibrillation: ROR = 15.06, 95% CI = 10.13–22.39; atrial flutter: ROR = 38.49, 95% CI = 15.98–92.69). This finding is further supported by a recent independent FAERS-based study, which also identified markedly elevated atrial fibrillation signals for ibrutinib-obinutuzumab combinations (ROR = 27.96) (Xiang et al., 2024), reinforcing the notion that BTKi-containing co-reported regimens warrant closer pharmacovigilance attention for elevated arrhythmogenic reporting signals. This finding is clinically important because BTKi-containing combination strategies are increasingly used to deepen responses in B-cell malignancies, including in elderly or less fit populations (Shanafelt et al., 2019; Tam et al., 2022; Zhu et al., 2022). Lenalidomide has been associated with vascular, thrombotic, and inflammatory toxicities, including endothelial dysfunction, oxidative stress, and pro-thrombotic effects (Maffei et al., 2016; Palumbo et al., 2008). We hypothesize that the combination of BTK/TEC pathway perturbation, BTKi-related electrophysiological vulnerability, and lenalidomide-associated endothelial or thrombo-inflammatory stress might collectively contribute to an elevated arrhythmogenic substrate. Crucially, because our analysis compared the co-listed group with the overall FAERS background rather than directly with BTKi alone or lenalidomide alone, and because a formal interaction analysis was not performed, we cannot determine whether this signal reflects a pharmacodynamic drug-drug interaction, confounding by patient characteristics, or stimulated reporting bias. They should therefore be regarded as hypothetical and exploratory, requiring validation in future experimental studies (e.g., electrophysiological assays, animal models, or targeted biomarker investigations) and well-designed prospective cohorts with formal interaction testing.

The association between higher body weight and cardiac adverse event reporting remained significant in multivariable analysis, suggesting that obesity or obesity-related cardiometabolic burden may contribute to increased reporting susceptibility during BTKi therapy. Importantly, because height and BMI data were unavailable in this FAERS-based analysis, higher body weight should not be equated with obesity, and this finding should be interpreted as reflecting reported weight rather than confirmed adiposity. While prior cardiovascular literature has established obesity as a risk factor for atrial fibrillation and adverse outcomes through mechanisms including atrial remodeling, systemic inflammation, hypertension, sleep apnea, and metabolic dysfunction (Aune et al., 2018; Powell-Wiley et al., 2021), these mechanistic insights can only be cautiously extrapolated to our weight-based observation, and further studies incorporating anthropometric measures are warranted to delineate the specific role of obesity. In the context of BTKi therapy, this characteristic may help identify patients potentially more susceptible to clinically manifest arrhythmias or other cardiac complications.

Based on these observed reporting signals, the following clinical considerations may be derived for cardio-oncology care. First, patients initiating acalabrutinib or zanubrutinib may warrant consideration of intensified early cardiovascular surveillance, particularly during the first 3 months of therapy, rather than relying solely on routine long-interval follow-up. Second, in light of the co-reporting signal observed for BTKi plus lenalidomide, baseline cardiovascular risk assessment, electrocardiography, blood pressure optimization, and consideration of cardiology consultation may be reasonable to consider. Third, patients with a reported body weight ≥80 kg (significantly associated with cardiac adverse event reporting in multivariable analysis) may warrant closer monitoring, though this should be interpreted as a reporting association, not as confirmed obesity or causality. Our recommendation for intensified ECG and cardiovascular risk surveillance aligns with and extends the 2022 ES C cardio-oncology guidelines, which emphasize baseline risk stratification, early detection of cancer therapy-related cardiovascular toxicity, and multidisciplinary management for patients receiving potentially cardiotoxic therapies (Lyon et al., 2022).

Compared with previous pharmacovigilance studies on BTK inhibitor-associated cardiotoxicity based on the FAERS database, the novelty of this study is mainly reflected in the following four aspects. First, we simultaneously employed four complementary disproportionality detection algorithms—ROR, PRR, BCPNN, and MGPS—with stringent concurrent threshold criteria, a conservative strategy that minimizes the inherent false-positive associations associated with single-algorithm designs. Second, within a unified analytical framework, we conducted a head-to-head comparison of cardiac event spectra across ibrutinib, acalabrutinib, and zanubrutinib, revealing that second-generation agents do not exhibit a disappearance of cardiac reporting signals but rather a shift in signal profiles relative to ibrutinib. Third, and most importantly from a clinical translational perspective, we provide the first systematic assessment of cardiac safety signals for modern BTKi-based co-reported regimens, including lenalidomide, venetoclax, and anti-CD20 monoclonal antibodies. Notably, the BTKi-lenalidomide co-reported subgroup showed the strongest atrial fibrillation and atrial flutter reporting signals among BTKi-related arrhythmias in the FAERS database (ROR up to 38.49), filling a critical gap in real-world safety data for such co-reported regiments. Fourth, we innovatively applied time-to-onset Weibull distribution modeling to define early reporting windows for cardiac adverse events, revealing a median reported time-to-onset of 66–72.5 days for second-generation BTKis. In parallel, we performed multidimensional phenotyping of reports with fatal outcomes to identify patient reporting characteristics associated with higher fatal reporting proportions, including male sex, age >85 years, and onset within 30 days. Together, these analyses translate raw pharmacovigilance data into clinically relevant monitoring strategies.

Several limitations should be acknowledged. FAERS is a spontaneous reporting system and is therefore subject to underreporting, duplicate reporting, stimulated reporting, missing data, and reporting bias. Although the FAERS database includes medication start and end dates (START_DT and END_DT) that could potentially be used to verify temporal overlapping exposure between drugs, these fields suffer from an exceedingly high rate of missing data—exceeding 80% in our dataset—along with a substantial number of inaccurate or imprecise entries. Consequently, we were unable to use these dates to confirm that BTKis and co-reported agents were actually administered concurrently. The Weber effect may influence the apparent temporal distribution of adverse events after drug approval. Moreover, FAERS lacks reliable denominators, precluding estimation of true incidence rates or absolute risks. Important clinical variables, including baseline cardiovascular disease, prior therapies, cancer stage, concomitant medications, electrocardiographic parameters, and laboratory data, are often unavailable. Specifically, regarding the interpretation of co-reported regimens, several potential confounding factors warrant consideration. Patients with co-listed BTKi and lenalidomide exposure may differ systematically from those on other regimens in terms of disease type (e.g., mantle cell lymphoma vs. diffuse large B-cell lymphoma), disease burden, prior lines of therapy, and baseline cardiovascular comorbidities—all of which could independently influence the reporting of cardiac arrhythmias. Furthermore, lenalidomide is known to be associated with thrombotic events and inflammatory responses, while the FAERS database lacks detailed concomitant medication records and laboratory data (e.g., D-dimer, C-reactive protein), precluding differentiation between pharmacodynamic drug-drug interactions and additive effects of underlying patient characteristics. Because our analysis compared the co-listed group with the overall FAERS background rather than directly with BTKi alone or lenalidomide alone, and because a formal interaction analysis was not performed, we cannot determine whether the observed signals reflect true pharmacodynamic interactions, confounding by indication, or stimulated reporting. Additionally, stimulated reporting bias cannot be excluded, as heightened clinical awareness of potential cardiotoxicity with novel co-reported regimens may inflate reporting rates in these subgroups. A further critical methodological consideration is that our combination analysis pooled all BTKis—including first-generation ibrutinib and second-generation acalabrutinib/zanubrutinib—as a single composite class. Given the fundamentally different baseline cardiotoxicity profiles between these agent generations, this approach may introduce substantial confounding. Indeed, ibrutinib-containing reports comprised the majority of the BTKi-lenalidomide subgroup (approximately 88%), making it impossible to determine whether the observed amplified signals reflect a true synergistic toxicity or are primarily driven by ibrutinib’s inherent cardiac risk. Consequently, the co-reported signals identified in this study should be interpreted as exploratory hypothesis-generating evidence rather than confirmatory causal associations. Future prospective cohort studies or registry-based analyses with adequate adjustment for baseline cardiovascular risk, disease characteristics, and concomitant therapies—ideally with sufficient sample sizes to stratify by individual BTKi agents—are warranted to validate whether the BTKi-lenalidomide safety signal is independent of these confounders and to formally test for pharmacodynamic interactions. Nevertheless, the consistency of cardiac disorder signals across multiple disproportionality algorithms, the concordance with major randomized trials, and the biologically plausible reporting phenotypes support the clinical relevance of these observations. Prospective cardio-oncology cohorts and registry-based studies are needed to validate these findings and determine optimal monitoring intervals for specific BTKi agents and co-reported regimens.

The therapeutic landscape of BTK inhibitors continues to evolve with the emergence of third-generation noncovalent agents, such as pirtobrutinib. Pirtobrutinib, a first-in-class noncovalent (reversible) BTK inhibitor, has shown promising efficacy in patients with B-cell malignancies progressing on covalent BTKis (Davis et al., 2024; Gomez et al., 2023; Keam, 2023; Mato et al., 2023; Naeem et al., 2023). Notably, emerging evidence suggests that pirtobrutinib may offer a favorable cardiovascular safety profile, with low rates of atrial fibrillation/flutter and hypertension reported in treatment-naïve patients (Mato et al., 2021; Mato et al., 2023; Wang et al., 2023). Future studies are warranted to determine the optimal role of third-generation BTKis in the treatment paradigm and to further define their cardiovascular safety profile compared with first- and second-generation agents in real-world populations.

## 5 Conclusion

This large-scale real-world pharmacovigilance study delineates distinct cardiac safety signal profiles across BTK inhibitors and BTKi-based co-reported regimens. While next-generation BTKis were associated with fewer positive cardiac signals than ibrutinib in our analysis, cardiac adverse events may be reported earlier after treatment initiation. The markedly enhanced atrial fibrillation and atrial flutter signals observed with BTKi-lenalidomide co-reported regimens highlight a clinically important safety signal requiring prospective validation. Recognition of reporting phenotypes associated with higher fatal reporting proportions, particularly very elderly male patients and those with very early cardiac symptoms, may help guide individualized cardio-oncology surveillance. These findings highlight the need for further investigation into early cardiovascular monitoring and multidisciplinary management to optimize the therapeutic index of BTKi therapy in patients with B-cell malignancies.

## Data Availability

Publicly available datasets were analyzed in this study. This data can be found here: The data are publicly available from the FAERS database (https://www.fda.gov/drugs).
