# Supplementary material for: Cardiac adverse event signals of Bruton’s tyrosine kinase inhibitors as primary suspect and in co-reported regimens: a large-scale FAERS pharmacovigilance study
Source: Front Pharmacol. 2026 Oct 14;17:1923976. doi: 10.3389/fphar.2026.1923976 (PMC13713257; doi:10.3389/fphar.2026.1923976)
Supplement: Supplementary file 1 [file Supplementaryfile1.docx]

**Supplementary Figures**

**Supplementary Figure 1.** Flowchart of adverse event report selection in this study.

**Supplementary Figure 2.** Baseline characteristics of adverse event reports for the three BTKi PS-based groups.

(A) Age. (B) Sex. (C) Body weight. (D) Reported outcomes: CA, congenital anomaly; DE, death; DS, disability; HO, hospitalization; LT, life-threatening; OT, other serious outcomes; RI, required intervention. (E) Time to AE occurrence. (F) Reporter occupation: CN, consumer; HP, health professional; LW, lawyer; MD, physician; OT, other health professional; PH, pharmacist; RN, registered nurse. (G) Reporting country. (H) Annual distribution of reports. From left to right: acalabrutinib, ibrutinib, and zanubrutinib.

**Supplementary Figure 3.** Baseline characteristics of adverse event (AE) reports for the three BTKi co-reported groups.

(A) Age. (B) Sex. (C) Body weight. (D) Reported outcomes: DE, death; DS, disability; HO, hospitalization; LT, life-threatening; OT, other serious outcomes. (E) Time to AE occurrence. (F) Reporter occupation: CN, consumer; HP, health professional; MD, physician; OT, other health professional; PH, pharmacist. (G) Reporting country. (H) Annual distribution of reports. From left to right: BTKi plus venetoclax, BTKi plus obinutuzumab/rituximab, and BTKi plus lenalidomide.

**Supplementary Figure 4.** Number of AE signals meeting the thresholds of all four signal detection algorithms at the Preferred Term level in the three BTKi PS-based groups.

(A) Acalabrutinib. (B) Ibrutinib. (C) Zanubrutinib.

**Supplementary Figure 5.** Number of AE signals meeting the thresholds of all four signal detection algorithms at the Preferred Term level in the three BTKi co-reported groups.

(A) BTKi plus venetoclax. (B) BTKi plus obinutuzumab/rituximab. (C) BTKi plus lenalidomide.

**Supplementary Figure 6.** Pharmacovigilance signal strength of cardiac adverse events across different reported groups.

(A) Reporting odds ratio (ROR). (B) Proportional reporting ratio (PRR). (C) Empirical Bayesian geometric mean (EBGM). (D) Information component (IC).
