# Supplementary material for: Cardiac adverse event signals of Bruton’s tyrosine kinase inhibitors as primary suspect and in co-reported regimens: a large-scale FAERS pharmacovigilance study
Source: Front Pharmacol. 2026 Oct 14;17:1923976. doi: 10.3389/fphar.2026.1923976 (PMC13713257; doi:10.3389/fphar.2026.1923976)
Supplement: Supplementary file 2 [file Table1.docx]

**Supplementary Table 1.** Drug name dictionaries for BTKis and co-reported agents

| Drugs | Search Terms |
| --- | --- |
| ibrutinib | 1-((3R)-3-(4-amino-3-(4-phenoxyphenyl)-1H-pyrazolo(3,4-d)pyrimidin-1-yl)piperidin-1-yl)prop-2-en-1-one; PCI 32765; PCI-32765; PCI32765; Imbruvica; Ibrutinib; IBRUTINIB; IBRUTINIB UNSPECIFIED; IBRUTINIB 140 MG PHARMACYCLICS JANSSEN; IMBRUVICA 140 MG PHARMACYCLICS |
| acalabrutinib | ACP-196; Calquence; Acalabrutinib; ACALABRUTINIB; ACP 196 |
| zanubrutinib | (7S)-2-(4-phenoxyphenyl)-7-(1-(prop-2-enoyl)piperidin-4-yl)-4,5,6,7-tetrahydropyrazolo(1,5-a)pyrimidine-3-carboxamide; BGB-3111; 7-(1-acryloyl-4-piperidinyl)-2-(4-phenoxyphenyl)-4,5,6,7-tetrahydropyrazolo(1,5-a)pyrimidine-3-carboxamide; Brukinsa; Zanubrutinib; ZANUBRUTINIB; BGB 3111 |
| anti-CD20 monoclonal antibodies (rituximab/obinutuzumab) | OBINUTUZUMAB; OBINUTUZUMAB MONOCLONAL ANTIBODIES UNKNOWN; OBINUTUZUMAB LIQUID; OBINUTUZUMAB GENETICAL RECOMBINATION; OBINUTUZUMAB SOLUTION FOR INFUSION; CHLORAMBUCIL OBINUTUZUMAB; OBINUTUZUMAB INFUSION; OBINUTUZUMAB 1000 MG GENENTECH; GAZYVA OBINUTUZUMAB; OBINUTUZUMAB OBINUTUZUMAB; GA101 OBINUTUZUMAB; OBINUTUZUMAB 1000MG; CHLORAMBUCIL W OBINUTUZUMAB; OBINUTUZUMAB OBINUTUZUMAB 25MG ML INJ VIL 40ML; OBINUTUZUMAB 1000MG; GAZYVA CHLORAMBUCIL OBINUTUZUMAB; OBINUTUZUMAB SOLUTION FOR INJECTION; OBINUTUZUMAB INV; BLINDED OBINUTUZUMAB; G BENDA OBINUTUZUMAB; OBINUTUZUMAB MONOCLONAL ANTIBODIES; GAZYVA; GAZYVARO; GAZYVA INTRAVENOUS SOLUTION 1000 MG; GAZYVARO 1000 MG; GAZYVA CHEMOTHERAPY; GAZYVA IMMUNOTHERAPY; GAZYVARO 1000 MG SOLUTION A DILUER POUR PERFUSION; GAZYVA INTRAVENOUS; AFUTUZUMAB; GA101; GA 101; RO5072759; RO 5072759; RO5072759 IGG1 MAB; RO 5072759 IGG1 MAB |
| venetoclax | VENETOCLAX 100MG; VENETOCLAX VENTETOCLAX 100MG TAB; VENETOCLAX 10 50 100MG DP; VENETOCLAX VENETOCLAX 10MGX14 50MGX7 100MGX21 STARTER PACK 21; VENETOCLAX VENETOCLAX 100MG TAB; VENETOCLAX VENETOCLAX 10MG TAB; VENETOCLAX 25MG TABS; VENETOCLAX VENCLEXTA; GDC 0199 BCL 2 SELECTIVE INHIBITOR; GDC 0199; GDC 0199 BCL 2 SELECTIVE INHIBITOR; GDC 0199 ABT 199; VENETOCLAX GDC 0199 ABT 199; VENCLEXTA; VENCLEXTA 100MG; VENCLEXTA STARTING PAC; VENCLEXTA 100MG ORAL; VENCLEXTA 100 MG TAB; VENETOCLAX VENCLEXTA; NOW VENCLEXTA FOR CLL; VENCLEXTA; ABT 199; GDC 0199 ABT 199; VENETOCLAX GDC 0199 ABT 199; ABT 199; ABT 199 VENETOCLAX OPEN LABEL; ABT 199 CLINICAL TRIAL DRUG; VENETCLAX ABT 199; VENETOXLAX ABT 199 |
| lenalidomide | LENALIDOMIDE; REVLIMID; CC 5013; CC5013; REVIMID |

**Supplementary Table 2.** Two-by-two contingency table used for signal detection

|  | Target adverse drug event | Other adverse drug event | Sums |
| --- | --- | --- | --- |
| Drug | a | b | a+b |
| Other drugs | c | d | c+d |
| Sums | a+c | b+d | a+b+c+d |

Note: a, number of reports in which the target drug and target AE were co-reported; b, number of reports involving the target drug and other AEs; c, number of reports involving the target AE and other drugs; d, number of reports involving other drugs and other AEs. AE, adverse event.

**Supplementary Table 3.** Overview of the signal detection algorithms used in this study

| Method | Equation | Criteria |
| --- | --- | --- |
| ROR | ROR=$\frac{\mathrm{ad}}{bc}$  ROR 95%CI=e^ln(ROR)±1.96^$\sqrt{\frac{1}{a}+\frac{1}{b}+\frac{1}{c}+\frac{1}{d}}$ | a ≥ 3 and 95%CI(lower limit) > 1 |
| PRR | PRR=$\frac{a(c+d)}{c(a+b)}$  χ2=[(ad-bc)^2](a+b+c+d)/[(a+b)(c+d)(a+c)(b+d)] | a ≥ 3, PRR ≥ 2, χ2 ≥ 4 |
| BCPNN | IC=log_2_$\frac{a(a+b+c+d)}{(a+c)(a+b)}$  95% CI = E(IC) ± 2[V(IC)]^0.5 | IC025 > 0 |
| MGPS | EBGM=$\frac{a(a+b+c+d)}{(a+c)(a+b)}$  EBGM05=e^ln(EBGM)±1.96^$\sqrt{\frac{1}{a}+\frac{1}{b}+\frac{1}{c}+\frac{1}{d}}$ | EBGM05 > 2 |

Abbreviation: ROR, reporting odds ratio; PRR, proportional reporting ratio; BCPNN, Bayesian confidence propagation neural network; MGPS, multi-item gamma Poisson shrinker; EBGM, empirical Bayesian geometric mean; 95% CI: 95% confidence interval; N: number of reports; χ2: chi-squared; IC: information component; IC025: lower limit of 95% CI of the IC; E (IC): IC expectations; V(IC): variance of IC; EBGM05: lower limit of 95% CI of EBGM.

**Supplementary Table 4.** FDA-approved Bruton's tyrosine kinase inhibitors (BTKis) and their inclusion in this study

| Drug | Approval time | Cases | Included in the study |
| --- | --- | --- | --- |
| Ibrutinib | 2013.11 | 67264 | Yes |
| Acalabrutinib | 2017.10 | 7906 | Yes |
| Zanubrutinib | 2019.11 | 1816 | Yes |
| Pirtobrutinib | 2023.01 | 448 | No |

**Supplementary Table 5.** Baseline characteristics of adverse event reports for BTKi PS-based groups

|  | All | Acalabrutinib | Ibrutinib | Zanubrutinib |
| --- | --- | --- | --- | --- |
|  | (N=76986) | (N=7906) | (N=67264) | (N=1816) |
| Gender |  |  |  |  |
| Female | 26543 (34.5%) | 2024 (25.6%) | 24502 (36.4%) | 17 (0.9%) |
| Male | 42016 (54.6%) | 3461 (43.8%) | 38521 (57.3%) | 34 (1.9%) |
| Unknown | 8427 (10.9%) | 2421 (30.6%) | 4241 (6.3%) | 1765 (97.2%) |
| Weight |  |  |  |  |
| ＜50 kg | 784 (1.0%) | 74 (0.9%) | 706 (1.0%) | 4 (0.2%) |
| 50～100 kg | 8980 (11.7%) | 1316 (16.6%) | 7615 (11.3%) | 49 (2.7%) |
| ＞100 kg | 1504 (2.0%) | 244 (3.1%) | 1254 (1.9%) | 6 (0.3%) |
| Unknown | 65718 (85.4%) | 6272 (79.3%) | 57689 (85.8%) | 1757 (96.8%) |
| Age |  |  |  |  |
| <18 | 385 (0.5%) | / | 385 (0.6%) | / |
| 18～64 | 7466 (9.7%) | 462 (5.8%) | 6974 (10.4%) | 30 (1.7%) |
| 65～85 | 22639 (29.4%) | 1838 (23.2%) | 20698 (30.8%) | 103 (5.7%) |
| >85 | 3347 (4.3%) | 386 (4.9%) | 2948 (4.4%) | 13 (0.7%) |
| Unknown | 43149 (56.0%) | 5220 (66.0%) | 36259 (53.9%) | 1670 (92.0%) |
| Reporter’s occupation |  |  |  |  |
| Consumer | 50387 (65.4%) | 4276 (54.1%) | 45441 (67.6%) | 670 (36.9%) |
| Health-professional | 6434 (8.4%) | 581 (7.3%) | 5573 (8.3%) | 280 (15.4%) |
| Lawyer | 5 (0.0%) | / | 5 (0.0%) | / |
| Physician | 13775 (17.9%) | 1739 (22.0%) | 11439 (17.0%) | 597 (32.9%) |
| Other health-professional | 3208 (4.2%) | 33 (0.4%) | 3175 (4.7%) | / |
| Pharmacist | 1926 (2.5%) | 219 (2.8%) | 1519 (2.3%) | 188 (10.4%) |
| Registered Nurse | 29 (0.0%) | / | 29 (0.0%) | / |
| Unknown | 1222 (1.6%) | 1058 (13.4%) | 83 (0.1%) | 81 (4.5%) |
| Outcome |  |  |  |  |
| Congenital anomaly | 6 (0.0%) | 3 (0.0%) | 2 (0.0%) | 1 (0.1%) |
| Death | 13552 (17.6%) | 2076 (26.3%) | 11310 (16.8%) | 166 (9.1%) |
| Disability | 202 (0.3%) | 38 (0.5%) | 153 (0.2%) | 11 (0.6%) |
| Hospitalization | 21028 (27.3%) | 1619 (20.5%) | 18941 (28.2%) | 468 (25.8%) |
| Life-threatening | 1117 (1.5%) | 184 (2.3%) | 897 (1.3%) | 36 (2.0%) |
| Other serious outcomes | 22020 (28.6%) | 2517 (31.8%) | 18947 (28.2%) | 556 (30.6%) |
| Required intervention | 19 (0.0%) | 6 (0.1%) | 10 (0.0%) | 3 (0.2%) |
| missing | 19042 (24.7%) | 1463 (18.5%) | 17004 (25.3%) | 575 (31.7%) |
| Top 5 countries |  |  |  |  |
|  | the United States  (56568, 73.5%) | the United States  (6199, 78.4%) | the United States  (49280, 73.3%) | the United States  (1089, 60.0) |
|  | Canada  (2140, 2.8%) | Canada  (322, 4.1%) | Canada  (1741, 2.6%) | China  (262, 14.4%) |
|  | France  (1615, 2.1%) | the United Kingdom  (211, 2.7%) | France  (1402, 2.1%) | Canada  (77, 4.2%) |
|  | Colombia  (1282, 1.7%) | France  (177, 2.2%) | Colombia  (1230, 1.8%) | Australia  (71, 3.9%) |
|  | the United Kingdom  (1189, 1.5%) | Germany  (135, 1.7%) | Germany  (973, 1.4%) | France  (36, 2.0%) |

**Supplementary Table 6.** Baseline characteristics of adverse event reports for BTKi co-reported regimens

|  | BTKis + BCL-2 inhibitor | BTKis + anti-CD20 monoclonal antibodies | BTKis + immunomodulatory agent |
| --- | --- | --- | --- |
|  |  |  |  |
|  | (N=1522) | (N=2803) | (N=251) |
| **Sex** |  |  |  |
| Female | 394 (25.9%) | 814 (29.0%) | 70 (27.9%) |
| Male | 891 (58.5%) | 1613 (57.5%) | 116 (46.2%) |
| Unknown | 237 (15.6%) | 376 (13.4%) | 65 (25.9%) |
| **Body Weight** |  |  |  |
| ＜50 kg | 7 (0.5%) | 57 (2.0%) | 6 (2.4%) |
| 50～100 kg | 50 (3.3%) | 146 (5.2%) | 10 (4.0%) |
| ＞100 kg | 272 (17.9%) | 833 (29.7%) | 43 (17.1%) |
| Unknown | 1193 (78.4%) | 1767 (63.0%) | 192 (76.5%) |
| **Age** |  |  |  |
| <18 | 17 (1.1%) | 31 (1.1%) | 1 (0.4%) |
| 18～64 | 25 (1.6%) | 63 (2.2%) | 5 (2.0%) |
| 65～85 | 318 (20.9%) | 717 (25.6%) | 59 (23.5%) |
| >85 | 498 (32.7%) | 1177 (42.0%) | 94 (37.5%) |
| Unknown | 664 (43.6%) | 815 (29.1%) | 92 (36.7%) |
| **Reporter occupation** |  |  |  |
| Consumer | 480 (31.5%) | 655 (23.4%) | 42 (16.7%) |
| Health-professional | 179 (11.8%) | 338 (12.1%) | 39 (15.5%) |
| Lawyer | / | / |  |
| Physician | 773 (50.8%) | 1614 (57.6%) | 144 (57.4%) |
| Other health-professional | 66 (4.3%) | 108 (3.9%) | 12 (4.8%) |
| Pharmacist | 14 (0.9%) | 45 (1.6%) | 10 (4.0%) |
| Registered Nurse | / | / |  |
| Unknown | 10 (0.7%) | 43 (1.5%) | 4 (1.6%) |
| **Reported outcomes** |  |  |  |
| Congenital anomaly | / | / | / |
| Death | 210 (13.8%) | 462 (16.5%) | 53 (21.1%) |
| Disability | 2 (0.1%) | 22 (0.8%) | / |
| Hospitalization | 575 (37.8%) | 1150 (41.0%) | 102 (40.6%) |
| Life-threatening | 49 (3.2%) | 179 (6.4%) | 8 (3.2%) |
| Other serious outcomes | 538 (35.3%) | 763 (27.2%) | 77 (30.7%) |
| Required intervention | / | / | / |
| unknown | 148 (9.7%) | 227 (8.1%) | 11 (4.4%) |
| **Top 5 reporting countries** |  |  |  |
|  | the United States (743, 48.8%) | the United States (1198, 42.7%) | the United States (124, 49.4%) |
|  | Australia (79, 5.2%) | France (157, 5.6%) | China (37, 14.7%) |
|  | Germany (76, 5.0%) | the United Kingdom (147, 5.2%) | France (16, 6.4%) |
|  | the United Kingdom (67, 4.4%) | Germany (140, 5.0%) | Sweden (12, 4.8%) |
|  | Spain (45, 3.0%) | China (133, 4.7%) | the United Kingdom (9, 3.6%) |

**Supplementary Table7.** Cardiac AE signals identified in the three BTKi PS-based groups

| **Groups** | **PT** | **N** | **SOC name** | **ROR(95%Cl)** | **PRR(Chi-square)** | **EBGM(EBGM05)** | **IC(IC025)** |
| --- | --- | --- | --- | --- | --- | --- | --- |
| **Ibrutinib** |  |  |  |  |  |  |  |
|  | ATRIAL FIBRILLATION | 2975 | CARDIAC DISORDERS | 9.86 ( 9.5 - 10.23 ) | 9.73 ( 22314.41 ) | 9.35 ( 9.06 ) | 3.22 ( 3.17 ) |
|  | CARDIAC DISORDER | 787 | CARDIAC DISORDERS | 2.77 ( 2.58 - 2.97 ) | 2.76 ( 873.88 ) | 2.74 ( 2.58 ) | 1.45 ( 1.35 ) |
|  | CARDIAC FAILURE CONGESTIVE | 534 | CARDIAC DISORDERS | 2.31 ( 2.12 - 2.51 ) | 2.3 ( 389.41 ) | 2.29 ( 2.13 ) | 1.19 ( 1.07 ) |
|  | PERICARDIAL EFFUSION | 397 | CARDIAC DISORDERS | 5.57 ( 5.04 - 6.16 ) | 5.56 ( 1448.1 ) | 5.45 ( 5.01 ) | 2.45 ( 2.3 ) |
|  | ARRHYTHMIA | 389 | CARDIAC DISORDERS | 2.8 ( 2.53 - 3.1 ) | 2.8 ( 443.74 ) | 2.77 ( 2.55 ) | 1.47 ( 1.32 ) |
|  | ATRIAL FLUTTER | 195 | CARDIAC DISORDERS | 8.23 ( 7.14 - 9.5 ) | 8.23 ( 1191.53 ) | 7.96 ( 7.06 ) | 2.99 ( 2.78 ) |
|  | VENTRICULAR TACHYCARDIA | 108 | CARDIAC DISORDERS | 2.38 ( 1.97 - 2.87 ) | 2.38 ( 85.25 ) | 2.36 ( 2.02 ) | 1.24 ( 0.96 ) |
|  | CARDIOTOXICITY | 87 | CARDIAC DISORDERS | 2.8 ( 2.27 - 3.46 ) | 2.8 ( 99.25 ) | 2.77 ( 2.32 ) | 1.47 ( 1.16 ) |
|  | CORONARY ARTERY OCCLUSION | 85 | CARDIAC DISORDERS | 2.49 ( 2.01 - 3.08 ) | 2.49 ( 74.8 ) | 2.47 ( 2.07 ) | 1.31 ( 0.99 ) |
|  | SUPRAVENTRICULAR TACHYCARDIA | 84 | CARDIAC DISORDERS | 3.11 ( 2.5 - 3.85 ) | 3.11 ( 118.18 ) | 3.07 ( 2.57 ) | 1.62 ( 1.31 ) |
|  | VENTRICULAR FIBRILLATION | 82 | CARDIAC DISORDERS | 2.78 ( 2.23 - 3.45 ) | 2.77 ( 91.86 ) | 2.75 ( 2.29 ) | 1.46 ( 1.14 ) |
|  | CARDIAC FLUTTER | 78 | CARDIAC DISORDERS | 3.77 ( 3.01 - 4.72 ) | 3.77 ( 155.98 ) | 3.72 ( 3.09 ) | 1.9 ( 1.57 ) |
|  | CARDIAC TAMPONADE | 63 | CARDIAC DISORDERS | 4.21 ( 3.28 - 5.4 ) | 4.21 ( 150.94 ) | 4.14 ( 3.36 ) | 2.05 ( 1.69 ) |
|  | PERICARDIAL HAEMORRHAGE | 54 | CARDIAC DISORDERS | 7.44 ( 5.67 - 9.76 ) | 7.44 ( 290.81 ) | 7.22 ( 5.75 ) | 2.85 ( 2.46 ) |
|  | SUPRAVENTRICULAR EXTRASYSTOLES | 40 | CARDIAC DISORDERS | 4.78 ( 3.5 - 6.54 ) | 4.78 ( 117.03 ) | 4.7 ( 3.61 ) | 2.23 ( 1.78 ) |
|  | VENTRICULAR ARRHYTHMIA | 38 | CARDIAC DISORDERS | 3.26 ( 2.37 - 4.49 ) | 3.26 ( 58.58 ) | 3.22 ( 2.47 ) | 1.69 ( 1.22 ) |
|  | SINUS NODE DYSFUNCTION | 32 | CARDIAC DISORDERS | 3.81 ( 2.69 - 5.41 ) | 3.81 ( 65.18 ) | 3.76 ( 2.81 ) | 1.91 ( 1.4 ) |
|  | ARRHYTHMIA SUPRAVENTRICULAR | 26 | CARDIAC DISORDERS | 9.68 ( 6.53 - 14.34 ) | 9.68 ( 193.44 ) | 9.3 ( 6.69 ) | 3.22 ( 2.65 ) |
|  | CARDIAC FIBRILLATION | 24 | CARDIAC DISORDERS | 5.72 ( 3.81 - 8.58 ) | 5.72 ( 91.01 ) | 5.6 ( 3.99 ) | 2.48 ( 1.9 ) |
|  | TACHYARRHYTHMIA | 22 | CARDIAC DISORDERS | 3.13 ( 2.06 - 4.77 ) | 3.13 ( 31.46 ) | 3.1 ( 2.18 ) | 1.63 ( 1.03 ) |
|  | AORTIC VALVE STENOSIS | 19 | CARDIAC DISORDERS | 3.51 ( 2.23 - 5.52 ) | 3.51 ( 33.52 ) | 3.47 ( 2.37 ) | 1.79 ( 1.14 ) |
|  | AORTIC VALVE DISEASE | 16 | CARDIAC DISORDERS | 3.33 ( 2.03 - 5.46 ) | 3.33 ( 25.74 ) | 3.3 ( 2.18 ) | 1.72 ( 1.02 ) |
|  | VENTRICULAR TACHYARRHYTHMIA | 13 | CARDIAC DISORDERS | 11.56 ( 6.61 - 20.2 ) | 11.56 ( 118.84 ) | 11.01 ( 6.9 ) | 3.46 ( 2.67 ) |
|  | PLEUROPERICARDITIS | 9 | CARDIAC DISORDERS | 7.68 ( 3.95 - 14.93 ) | 7.68 ( 50.43 ) | 7.44 ( 4.27 ) | 2.9 ( 1.97 ) |
|  | SINOATRIAL BLOCK | 7 | CARDIAC DISORDERS | 4.43 ( 2.1 - 9.36 ) | 4.43 ( 18.2 ) | 4.36 ( 2.33 ) | 2.12 ( 1.09 ) |
|  | ARRHYTHMIC STORM | 5 | CARDIAC DISORDERS | 4.58 ( 1.89 - 11.11 ) | 4.58 ( 13.69 ) | 4.5 ( 2.15 ) | 2.17 ( 0.98 ) |
|  | HEART VALVE CALCIFICATION | 4 | CARDIAC DISORDERS | 6.8 ( 2.51 - 18.4 ) | 6.8 ( 19.16 ) | 6.62 ( 2.88 ) | 2.73 ( 1.41 ) |
|  | TACHYCARDIA PAROXYSMAL | 4 | CARDIAC DISORDERS | 5.08 ( 1.88 - 13.69 ) | 5.08 ( 12.79 ) | 4.98 ( 2.17 ) | 2.32 ( 1.01 ) |
|  | BIFASCICULAR BLOCK | 3 | CARDIAC DISORDERS | 5.96 ( 1.89 - 18.79 ) | 5.96 ( 12.05 ) | 5.83 ( 2.23 ) | 2.54 ( 1.07 ) |
| **Acalabrutinib** |  |  |  |  |  |  |  |
|  | ATRIAL FIBRILLATION | 169 | CARDIAC DISORDERS | 6.05 ( 5.2 - 7.04 ) | 6 ( 703.34 ) | 5.99 ( 5.27 ) | 2.58 ( 2.36 ) |
|  | MYOCARDIAL INFARCTION | 83 | CARDIAC DISORDERS | 3 ( 2.42 - 3.72 ) | 2.99 ( 109.78 ) | 2.98 ( 2.49 ) | 1.58 ( 1.26 ) |
|  | CARDIAC DISORDER | 76 | CARDIAC DISORDERS | 3.11 ( 2.49 - 3.9 ) | 3.11 ( 108.49 ) | 3.1 ( 2.57 ) | 1.63 ( 1.3 ) |
|  | CARDIAC FAILURE CONGESTIVE | 40 | CARDIAC DISORDERS | 3.4 ( 2.49 - 4.64 ) | 3.4 ( 67.53 ) | 3.39 ( 2.62 ) | 1.76 ( 1.31 ) |
|  | CARDIOTOXICITY | 39 | CARDIAC DISORDERS | 12.27 ( 8.95 - 16.82 ) | 12.24 ( 399.95 ) | 12.17 ( 9.34 ) | 3.6 ( 3.15 ) |
|  | PERICARDIAL EFFUSION | 24 | CARDIAC DISORDERS | 3.74 ( 2.51 - 5.59 ) | 3.74 ( 48.04 ) | 3.73 ( 2.67 ) | 1.9 ( 1.32 ) |
|  | ANGINA PECTORIS | 21 | CARDIAC DISORDERS | 3.12 ( 2.03 - 4.79 ) | 3.12 ( 30.22 ) | 3.12 ( 2.18 ) | 1.64 ( 1.02 ) |
|  | VENTRICULAR EXTRASYSTOLES | 12 | CARDIAC DISORDERS | 5.32 ( 3.02 - 9.37 ) | 5.31 ( 41.89 ) | 5.3 ( 3.3 ) | 2.41 ( 1.6 ) |
|  | CARDIAC FLUTTER | 10 | CARDIAC DISORDERS | 5.59 ( 3.01 - 10.41 ) | 5.59 ( 37.59 ) | 5.58 ( 3.32 ) | 2.48 ( 1.61 ) |
|  | ATRIAL FLUTTER | 8 | CARDIAC DISORDERS | 3.91 ( 1.95 - 7.82 ) | 3.91 ( 17.27 ) | 3.9 ( 2.18 ) | 1.96 ( 1 ) |
|  | SUPRAVENTRICULAR EXTRASYSTOLES | 6 | CARDIAC DISORDERS | 8.51 ( 3.82 - 18.99 ) | 8.51 ( 39.57 ) | 8.47 ( 4.33 ) | 3.08 ( 1.99 ) |
|  | ATRIAL TACHYCARDIA | 5 | CARDIAC DISORDERS | 8.15 ( 3.38 - 19.62 ) | 8.15 ( 31.2 ) | 8.11 ( 3.89 ) | 3.02 ( 1.84 ) |
|  | CARDIAC DISCOMFORT | 4 | CARDIAC DISORDERS | 5.72 ( 2.14 - 15.27 ) | 5.72 ( 15.53 ) | 5.71 ( 2.51 ) | 2.51 ( 1.22 ) |
|  | AORTIC VALVE STENOSIS | 3 | CARDIAC DISORDERS | 5.97 ( 1.92 - 18.56 ) | 5.97 ( 12.38 ) | 5.96 ( 2.31 ) | 2.57 ( 1.13 ) |
|  | CARDIAC AMYLOIDOSIS | 3 | CARDIAC DISORDERS | 6.79 ( 2.19 - 21.11 ) | 6.79 ( 14.76 ) | 6.77 ( 2.62 ) | 2.76 ( 1.31 ) |
|  | CARDIAC FIBRILLATION | 3 | CARDIAC DISORDERS | 8.85 ( 2.85 - 27.54 ) | 8.85 ( 20.79 ) | 8.81 ( 3.41 ) | 3.14 ( 1.69 ) |
| **Zanubrutinib** |  |  |  |  |  |  |  |
|  | ATRIAL FIBRILLATION | 44 | CARDIAC DISORDERS | 5.23 ( 3.88 - 7.03 ) | 5.19 ( 149.08 ) | 5.19 ( 4.05 ) | 2.38 ( 1.94 ) |
|  | CARDIAC FAILURE | 20 | CARDIAC DISORDERS | 2.94 ( 1.89 - 4.56 ) | 2.93 ( 25.47 ) | 2.93 ( 2.03 ) | 1.55 ( 0.92 ) |
|  | PERICARDIAL EFFUSION | 11 | CARDIAC DISORDERS | 5.75 ( 3.18 - 10.39 ) | 5.74 ( 43.04 ) | 5.74 ( 3.5 ) | 2.52 ( 1.69 ) |
|  | VENTRICULAR TACHYCARDIA | 5 | CARDIAC DISORDERS | 4.21 ( 1.75 - 10.12 ) | 4.2 ( 12.2 ) | 4.2 ( 2.02 ) | 2.07 ( 0.89 ) |
|  | CARDIAC FLUTTER | 4 | CARDIAC DISORDERS | 7.61 ( 2.85 - 20.3 ) | 7.6 ( 22.9 ) | 7.59 ( 3.34 ) | 2.92 ( 1.63 ) |
|  | CARDIAC TAMPONADE | 4 | CARDIAC DISORDERS | 10.6 ( 3.97 - 28.29 ) | 10.59 ( 34.67 ) | 10.57 ( 4.65 ) | 3.4 ( 2.11 ) |
|  | ATRIAL FLUTTER | 3 | CARDIAC DISORDERS | 5.3 ( 1.71 - 16.45 ) | 5.3 ( 10.45 ) | 5.29 ( 2.05 ) | 2.4 ( 0.96 ) |

**Supplementary Table 8.** Cardiac AE signals identified in the BTKi co-reported groups

| **Groups** | **PT** | **N** | **SOC name** | **ROR(95%Cl)** | **PRR(Chi-square)** | **EBGM(EBGM05)** | **IC(IC025)** |
| --- | --- | --- | --- | --- | --- | --- | --- |
| **BTKis + obinutuzumab/rituximab** |  |  |  |  |  |  |  |
|  | ATRIAL FIBRILLATION | 184 | CARDIAC DISORDERS | 11.18 ( 9.67 - 12.94 ) | 11.02 ( 1673.43 ) | 10.99 ( 9.73 ) | 3.46 ( 3.24 ) |
|  | CARDIAC FAILURE | 39 | CARDIAC DISORDERS | 2.85 ( 2.08 - 3.91 ) | 2.85 ( 46.76 ) | 2.85 ( 2.19 ) | 1.51 ( 1.05 ) |
|  | PERICARDIAL EFFUSION | 23 | CARDIAC DISORDERS | 6.01 ( 3.99 - 9.06 ) | 6 ( 95.81 ) | 6 ( 4.26 ) | 2.58 ( 1.99 ) |
|  | ATRIAL FLUTTER | 23 | CARDIAC DISORDERS | 17.94 ( 11.91 - 27.04 ) | 17.91 ( 365.6 ) | 17.83 ( 12.65 ) | 4.16 ( 3.57 ) |
|  | PERICARDITIS | 13 | CARDIAC DISORDERS | 4.32 ( 2.51 - 7.45 ) | 4.32 ( 33.15 ) | 4.32 ( 2.74 ) | 2.11 ( 1.34 ) |
|  | SUPRAVENTRICULAR TACHYCARDIA | 11 | CARDIAC DISORDERS | 7.67 ( 4.25 - 13.87 ) | 7.67 ( 63.65 ) | 7.65 ( 4.66 ) | 2.94 ( 2.1 ) |
|  | SINUS TACHYCARDIA | 10 | CARDIAC DISORDERS | 4.78 ( 2.57 - 8.89 ) | 4.78 ( 29.85 ) | 4.77 ( 2.84 ) | 2.26 ( 1.38 ) |
|  | VENTRICULAR TACHYCARDIA | 9 | CARDIAC DISORDERS | 3.75 ( 1.95 - 7.2 ) | 3.74 ( 18.09 ) | 3.74 ( 2.16 ) | 1.9 ( 0.99 ) |
|  | ACUTE CORONARY SYNDROME | 9 | CARDIAC DISORDERS | 6.77 ( 3.52 - 13.02 ) | 6.76 ( 44.12 ) | 6.75 ( 3.91 ) | 2.76 ( 1.84 ) |
|  | VENTRICULAR FIBRILLATION | 7 | CARDIAC DISORDERS | 4.47 ( 2.13 - 9.38 ) | 4.47 ( 18.82 ) | 4.46 ( 2.4 ) | 2.16 ( 1.14 ) |
|  | SUPRAVENTRICULAR EXTRASYSTOLES | 7 | CARDIAC DISORDERS | 15.7 ( 7.47 - 32.98 ) | 15.69 ( 95.88 ) | 15.63 ( 8.4 ) | 3.97 ( 2.94 ) |
|  | SINUS BRADYCARDIA | 6 | CARDIAC DISORDERS | 3.96 ( 1.78 - 8.81 ) | 3.95 ( 13.23 ) | 3.95 ( 2.02 ) | 1.98 ( 0.89 ) |
|  | ATRIOVENTRICULAR BLOCK | 6 | CARDIAC DISORDERS | 4.9 ( 2.2 - 10.92 ) | 4.9 ( 18.6 ) | 4.89 ( 2.5 ) | 2.29 ( 1.2 ) |
|  | LEFT VENTRICULAR FAILURE | 3 | CARDIAC DISORDERS | 5.68 ( 1.83 - 17.62 ) | 5.68 ( 11.54 ) | 5.67 ( 2.2 ) | 2.5 ( 1.06 ) |
|  | ATRIAL TACHYCARDIA | 3 | CARDIAC DISORDERS | 8.82 ( 2.84 - 27.38 ) | 8.81 ( 20.74 ) | 8.8 ( 3.41 ) | 3.14 ( 1.69 ) |
| **BTKis + venetoclax** |  |  |  |  |  |  |  |
|  | ATRIAL FIBRILLATION | 127 | CARDIAC DISORDERS | 13.27 ( 11.13 - 15.82 ) | 13.03 ( 1409.55 ) | 13 ( 11.22 ) | 3.7 ( 3.44 ) |
|  | ARRHYTHMIA | 22 | CARDIAC DISORDERS | 5.13 ( 3.37 - 7.8 ) | 5.12 ( 72.85 ) | 5.11 ( 3.6 ) | 2.35 ( 1.75 ) |
|  | ATRIAL FLUTTER | 13 | CARDIAC DISORDERS | 17.36 ( 10.07 - 29.93 ) | 17.32 ( 199.49 ) | 17.28 ( 10.96 ) | 4.11 ( 3.34 ) |
|  | SUPRAVENTRICULAR TACHYCARDIA | 6 | CARDIAC DISORDERS | 7.17 ( 3.22 - 15.97 ) | 7.16 ( 31.79 ) | 7.16 ( 3.66 ) | 2.84 ( 1.75 ) |
|  | VENTRICULAR FIBRILLATION | 5 | CARDIAC DISORDERS | 5.47 ( 2.28 - 13.16 ) | 5.47 ( 18.26 ) | 5.47 ( 2.62 ) | 2.45 ( 1.27 ) |
|  | VENTRICULAR ARRHYTHMIA | 4 | CARDIAC DISORDERS | 11.08 ( 4.15 - 29.56 ) | 11.08 ( 36.61 ) | 11.06 ( 4.87 ) | 3.47 ( 2.18 ) |
|  | SINUS NODE DYSFUNCTION | 3 | CARDIAC DISORDERS | 11.51 ( 3.71 - 35.74 ) | 11.51 ( 28.74 ) | 11.49 ( 4.45 ) | 3.52 ( 2.08 ) |
| **BTKis + lenalidomide** |  |  |  |  |  |  |  |
|  | ATRIAL FIBRILLATION | 25 | CARDIAC DISORDERS | 15.06 ( 10.13 - 22.39 ) | 14.75 ( 320.74 ) | 14.74 ( 10.58 ) | 3.88 ( 3.31 ) |
|  | CARDIAC ARREST | 8 | CARDIAC DISORDERS | 6.44 ( 3.21 - 12.92 ) | 6.41 ( 36.53 ) | 6.41 ( 3.58 ) | 2.68 ( 1.71 ) |
|  | VENTRICULAR FIBRILLATION | 5 | CARDIAC DISORDERS | 31.65 ( 13.14 - 76.21 ) | 31.51 ( 147.62 ) | 31.49 ( 15.09 ) | 4.98 ( 3.8 ) |
|  | ATRIAL FLUTTER | 5 | CARDIAC DISORDERS | 38.49 ( 15.98 - 92.69 ) | 38.32 ( 181.57 ) | 38.28 ( 18.35 ) | 5.26 ( 4.08 ) |

**Supplementary Table 9.** Deduplicated list of cardiac AEs across all reported groups

| **PT** | **SOC name** | **HLT name** | **HLGT name** |
| --- | --- | --- | --- |
| Sinus Node Dysfunction | Cardiac Disorders | Cardiac Arrhythmias | Supraventricular Arrhythmias |
| Myocardial Infarction | Cardiac Disorders | Coronary Artery Disorders | Ischaemic Coronary Artery Disorders |
| Supraventricular Extrasystoles | Cardiac Disorders | Cardiac Arrhythmias | Supraventricular Arrhythmias |
| Heart Valve Incompetence | Cardiac Disorders | Cardiac Valve Disorders | Cardiac Valve Disorders Nec |
| Ventricular Tachycardia | Cardiac Disorders | Cardiac Arrhythmias | Ventricular Arrhythmias And Cardiac Arrest |
| Ventricular Tachyarrhythmia | Cardiac Disorders | Cardiac Arrhythmias | Ventricular Arrhythmias And Cardiac Arrest |
| Ventricular Fibrillation | Cardiac Disorders | Cardiac Arrhythmias | Ventricular Arrhythmias And Cardiac Arrest |
| Ventricular Extrasystoles | Cardiac Disorders | Cardiac Arrhythmias | Ventricular Arrhythmias And Cardiac Arrest |
| Ventricular Arrhythmia | Cardiac Disorders | Cardiac Arrhythmias | Ventricular Arrhythmias And Cardiac Arrest |
| Tachycardia Paroxysmal | Cardiac Disorders | Cardiac Arrhythmias | Rate And Rhythm Disorders Nec |
| Tachyarrhythmia | Cardiac Disorders | Cardiac Arrhythmias | Rate And Rhythm Disorders Nec |
| Supraventricular Tachycardia | Cardiac Disorders | Cardiac Arrhythmias | Supraventricular Arrhythmias |
| Sinus Tachycardia | Cardiac Disorders | Cardiac Arrhythmias | Supraventricular Arrhythmias |
| Sinus Bradycardia | Cardiac Disorders | Cardiac Arrhythmias | Supraventricular Arrhythmias |
| Sinoatrial Block | Cardiac Disorders | Cardiac Arrhythmias | Cardiac Conduction Disorders |
| Pleuropericarditis | Cardiac Disorders | Pericardial Disorders | Noninfectious Pericarditis |
| Pericarditis | Cardiac Disorders | Pericardial Disorders | Noninfectious Pericarditis |
| Pericardial Haemorrhage | Cardiac Disorders | Pericardial Disorders | Pericardial Disorders Nec |
| Pericardial Effusion | Cardiac Disorders | Pericardial Disorders | Pericardial Disorders Nec |
| Left Ventricular Failure | Cardiac Disorders | Heart Failures | Left Ventricular Failures |
| Heart Valve Calcification | Cardiac Disorders | Cardiac Valve Disorders | Cardiac Valve Disorders Nec |
| Coronary Artery Occlusion | Cardiac Disorders | Coronary Artery Disorders | Coronary Artery Disorders Nec |
| Cardiotoxicity | Cardiac Disorders | Cardiac Disorders, Signs And Symptoms Nec | Cardiac Disorders Nec |
| Cardiac Tamponade | Cardiac Disorders | Pericardial Disorders | Pericardial Disorders Nec |
| Cardiac Flutter | Cardiac Disorders | Cardiac Arrhythmias | Rate And Rhythm Disorders Nec |
| Cardiac Fibrillation | Cardiac Disorders | Cardiac Arrhythmias | Rate And Rhythm Disorders Nec |
| Cardiac Failure Congestive | Cardiac Disorders | Heart Failures | Heart Failures Nec |
| Cardiac Failure | Cardiac Disorders | Heart Failures | Heart Failures Nec |
| Cardiac Disorder | Cardiac Disorders | Cardiac Disorders, Signs And Symptoms Nec | Cardiac Disorders Nec |
| Cardiac Discomfort | Cardiac Disorders | Cardiac Disorders, Signs And Symptoms Nec | Cardiac Signs And Symptoms Nec |
| Cardiac Arrest | Cardiac Disorders | Cardiac Arrhythmias | Ventricular Arrhythmias And Cardiac Arrest |
| Cardiac Amyloidosis | Cardiac Disorders | Myocardial Disorders | Cardiomyopathies |
| Bifascicular Block | Cardiac Disorders | Cardiac Arrhythmias | Cardiac Conduction Disorders |
| Atrioventricular Block | Cardiac Disorders | Cardiac Arrhythmias | Cardiac Conduction Disorders |
| Atrial Tachycardia | Cardiac Disorders | Cardiac Arrhythmias | Supraventricular Arrhythmias |
| Atrial Flutter | Cardiac Disorders | Cardiac Arrhythmias | Supraventricular Arrhythmias |
| Atrial Fibrillation | Cardiac Disorders | Cardiac Arrhythmias | Supraventricular Arrhythmias |
| Arrhythmic Storm | Cardiac Disorders | Cardiac Arrhythmias | Ventricular Arrhythmias And Cardiac Arrest |
| Arrhythmia Supraventricular | Cardiac Disorders | Cardiac Arrhythmias | Supraventricular Arrhythmias |
| Arrhythmia | Cardiac Disorders | Cardiac Arrhythmias | Rate And Rhythm Disorders Nec |
| Aortic Valve Thickening | Cardiac Disorders | Cardiac Valve Disorders | Aortic Valvular Disorders |
| Aortic Valve Stenosis | Cardiac Disorders | Cardiac Valve Disorders | Aortic Valvular Disorders |
| Aortic Valve Disease | Cardiac Disorders | Cardiac Valve Disorders | Aortic Valvular Disorders |
| Angina Pectoris | Cardiac Disorders | Coronary Artery Disorders | Ischaemic Coronary Artery Disorders |
| Acute Coronary Syndrome | Cardiac Disorders | Coronary Artery Disorders | Ischaemic Coronary Artery Disorders |

**Supplementary Table 10.** Weibull analysis of time-to-onset for cardiac AEs in the BTKi PS-based groups

| Drug | Case | TTO (days) | | Weibull distribution | | | | Failure type |
| --- | --- | --- | --- | --- | --- | --- | --- | --- |
|  |  |  |  | Scale parameter | | Shape parameter | |  |
|  |  | Media(IQR) | Min~Max | α | 95% CI | β | 95% CI |  |
| Acalabrutinib | 140 | 72.5(15-182.75) | 1~1154 | 119.74 | 92.51~146.96 | 0.65 | 0.58~0.72 | Early failure |
| Ibrutinib | 2221 | 150(42-479) | 1~2842 | 283.59 | 268.75~298.42 | 0.7 | 0.69~0.72 | Early failure |
| Zanubrutinib | 54 | 66(16.5-222.5) | 2~1373 | 132.92 | 83.76~182.07 | 0.64 | 0.53~0.75 | Early failure |

**Supplementary Table 11.** Weibull analysis of time-to-onset for cardiac AEs in the BTKi co-reported groups

| Drug | Case | TTO (days) | | Weibull distribution | | | | Failure type |
| --- | --- | --- | --- | --- | --- | --- | --- | --- |
|  |  |  |  | Scale parameter | | Shape parameter | |  |
|  |  | Media(IQR) | Min~Max | α | 95% CI | β | 95% CI |  |
| BTK_Ven | 70 | 180.5(65.5-371) | 2~1613 | 253.92 | 196.66~311.18 | 0.92 | 0.78~1.06 | Random failure |
| BTK_CD20 | 151 | 81(33-292) | 1~1941 | 189.06 | 148.93~229.19 | 0.67 | 0.6~0.74 | Early failure |
| BTK_Len | 19 | 34(7-175) | 1~812 | 94.83 | 25.14~164.51 | 0.54 | 0.38~0.7 | Early failure |

**Supplementary Table 12.** Baseline characteristics of cardiac adverse event reports stratified by fatal outcome status

|  | **Fatal** | **Non-fatal** | **Overall** |
| --- | --- | --- | --- |
|  | (N=1125) | (N=7141) | (N=8266) |
| **Sex** |  |  |  |
| Female | 224 (19.9%) | 2156 (30.2%) | 2380 (28.8%) |
| Male | 700 (62.2%) | 3969 (55.6%) | 4669 (56.5%) |
| Unknown | 201 (17.9%) | 1016 (14.2%) | 1217 (14.7%) |
| **Body Weight** |  |  |  |
| < 50 kg | 29 (2.6%) | 49 (0.7%) | 78 (0.9%) |
| > 100 kg | 41 (3.6%) | 235 (3.3%) | 276 (3.3%) |
| 50～100 kg | 247 (22.0%) | 1029 (14.4%) | 1276 (15.4%) |
| Unknown | 808 (71.8%) | 5828 (81.6%) | 6636 (80.3%) |
| **Age** |  |  |  |
| < 18 | 6 (0.5%) | 36 (0.5%) | 42 (0.5%) |
| > 85 | 105 (9.3%) | 307 (4.3%) | 412 (5.0%) |
| 18～64 | 140 (12.4%) | 691 (9.7%) | 831 (10.1%) |
| 65～85 | 448 (39.8%) | 2717 (38.0%) | 3165 (38.3%) |
| Unknown | 426 (37.9%) | 3390 (47.5%) | 3816 (46.2%) |
| **Reporter occupation** |  |  |  |
| Consumer | 339 (30.1%) | 3958 (55.4%) | 4297 (52.0%) |
| Health-professional | 183 (16.3%) | 545 (7.6%) | 728 (8.8%) |
| Physician | 433 (38.5%) | 1888 (26.4%) | 2321 (28.1%) |
| Other health-professional | 120 (10.7%) | 402 (5.6%) | 522 (6.3%) |
| Pharmacist | 34 (3.0%) | 241 (3.4%) | 275 (3.3%) |
| Registered Nurse | / | 2 (0.0%) | 2 (0.0%) |
| Unknown | 16 (1.4%) | 105 (1.5%) | 121 (1.5%) |
| **Time_group** |  |  |  |
| 0-30 days | 77 (6.8%) | 297 (4.2%) | 374 (4.5%) |
| 31-60 days | 42 (3.7%) | 158 (2.2%) | 200 (2.4%) |
| 61-90 days | 24 (2.1%) | 118 (1.7%) | 142 (1.7%) |
| 91-120 days | 15 (1.3%) | 91 (1.3%) | 106 (1.3%) |
| 121-150 days | 8 (0.7%) | 74 (1.0%) | 82 (1.0%) |
| 151-180 days | 12 (1.1%) | 55 (0.8%) | 67 (0.8%) |
| 181-360 days | 47 (4.2%) | 224 (3.1%) | 271 (3.3%) |
| >360 days | 73 (6.5%) | 472 (6.6%) | 545 (6.6%) |
| Unknown | 827 (73.5%) | 5652 (79.1%) | 6479 (78.4%) |
| **Drug** |  |  |  |
| Acalabrutinib | 53 (4.7%) | 665 (9.3%) | 718 (8.7%) |
| Ibrutinib | 1053 (93.6%) | 6351 (88.9%) | 7404 (89.6%) |
| Zanubrutinib | 19 (1.7%) | 125 (1.8%) | 144 (1.7%) |

**Supplementary Table 13.** Collinearity diagnostics of covariates included in the logistic regression models

| Covariates | GVIF | Df | GVIF^(1/(2*Df)) |
| --- | --- | --- | --- |
| Age | 1.028725 | 1 | 1.014261 |
| Weight | 1.165253 | 1 | 1.079469 |
| Sex | 1.141591 | 1 | 1.068453 |
| Drug | 1.012387 | 2 | 1.003082 |
